# γ-Aminobutyric Acid Promotes Chloroplast Ultrastructure, Antioxidant Capacity, and Growth of Waterlogged Maize Seedlings

**DOI:** 10.1038/s41598-018-36334-y

**Published:** 2019-01-24

**Authors:** Akram Salah, Ming Zhan, Cougui Cao, Yuling Han, Lin Ling, Zhihui Liu, Ping Li, Miao Ye, Yang Jiang

**Affiliations:** MOA Key Laboratory of Crop Physiology, Ecology and Cultivation in the Middle Reaches of Yangtze River, College of Plant Science and Technology, Huazhong Agricultural University, Wuhan, 430070 China

## Abstract

γ-aminobutyric acid (GABA) is a small signaling molecule that accumulates rapidly in plants exposed to various stresses; however, it has not been applied in regulating waterlogging tolerance in maize seedlings. Here, the effect of exogenous application of GABA in the determined optimal concentration was performed on seedlings of two maize cultivars under waterlogging treatments initiated at 3-leaf (V3) and 5-leaf stages (V5) in repeated experiments in 2016 and 2017. Chloroplast ultrastructure, photosynthesis, antioxidant capacity, and reactive oxygen species (ROS) production in the leaves were examined and compared with the corresponding values under normal soil water treatment (CK) and waterlogging treatment (WL). Compared with WL treatment, application of GABA significantly increased aboveground and root dry matter by 19.0% and 61.0%, promoted photosynthetic rate and chlorophyll content by 19.8% and 36.0%, increased the number of grana per chloroplast by 36.0%, fortified antioxidants (SOD, POD, CAT, GR, APX, V_C_) activities by 14.7–42.7%, and reduced the content of MDA, H_2_O_2,_ and O_2_^−^ by 30.5%, 32.5%, and 21.8%, respectively (p < 0.05). Collectively, GABA application was shown to promote the growth of maize seedlings under waterlogging, by down regulating ROIs-producing enzymes, activating antioxidant defense systems, and improving chloroplast ultrastructure and photosynthetic traits.

## Introduction

Globally, maize (*Zea mays L*.) is one of the most important cereal crops of tropical and subtropical environments. Like other crops, maize is more sensitive to waterlogging, particularly at the seedling stage, which is considered one of the major threats to the production of this crop^1,2^. In the tropical and subtropical region of China, most rainfall occurs in the maize growing season and excessive rainfall and flooding has caused heavy loss of crop growth and yield^3,4^. Global climate change contributes to an increasingly unpredictable rain pattern and is causing flooding that is expected to increase crop yield losses^5^. Globally, it is estimated that 12% of cultivated areas are affected by waterlogging, resulting in a 20% decrease in crop production^6,7^ and increasing annual losses to billions of dollars^8^. Waterlogging depletes the oxygen in soil because of slow diffusion and high consumption by plant roots^9,10^. When maize crop is subjected to waterlogging, stomatal closure and declines in photosynthetic rates are common responses^11,12^. Excessive soil moisture limits plant growth by altering morphological, physiological, and anatomical mechanisms^13–15^. Waterlogging decreases superoxide dismutase (SOD), peroxidase (POD), ascorbate peroxidase (APX) and catalase (CAT) enzyme activities, causing damage to the protective enzyme system. Waterlogging also regulates the malondialdehyde (MDA) content, resulting in membrane deterioration, leaf chlorosis, and increased leaf senescence^16–18^.

Chloroplasts are the major sites for generating reactive oxygen species (ROS) under environmental stress conditions^19^. Accumulation of excessive ROS could lead to oxidation of biomolecules, and such processes trigger oxidative damages to proteins, nucleic acids, and lipids and peroxidation of thylakoid membrane lipids^20^. Previous researchers showed that waterlogging stress changed the shape and internal structure of chloroplasts and their volume, and also damage the structure and integrity of cell membrane and mitochondria^1^. The physiological reaction to waterlogging results in reduced leaf area, chlorophyll content, chlorophyll fluorescence, and destruction of chloroplast ultrastructure in maize^1^. The structure of mesophyll cell and chloroplast morphology is an essential component of photosynthesis and plays a key role in determining photosynthetic assimilation capacity^21^. The morphology and ultrastructure of chloroplasts directly affects photosynthesis and significantly reduces the dry matter accumulation and yield of maize crop^1,2,6–8,13,15–17,19–22^.

Plant growth regulators play an important role in modulating plant physiological responses to adapt to an unfavorable environment^23^, and their exogenous application is an effective way to improve crop tolerance to waterlogging stress. Aminobutyric acid (GABA) is an important four- carbon, non-protein amino acid present in animals, plants, and other organisms, and is involved in certain physio-biochemical functions for the regulation of plant growth and stress tolerance, such as signaling, regulation of redox status, sustenance of cytosolic pH, osmotic pressure, C and N metabolism, and C-N fluxes^24–26^. Current studies indicate that GABA responds rapidly to abiotic stresses such as waterlogging, salt stress, heat shock, low temperature, mechanical stimulation, and plant hormones^27–31^. Moreover, exogenous application of GABA regulates the gene expressions of NO_3_^−^ uptake and NO_3_^−^ transport (*BnNrt2*) in the *Brassica napus*^32^ and *14-3-3* in *Arabidopsis thaliana*^33^. It has also been reported that gene regulation in GABA application is associated with H_2_O_2_ and ethylene production in the roots of caragana^34^. GABA plays a critical role in alleviating oxidative damage through activation of the antioxidant enzymes, which constitute a defense system against ROS in wheat plants under waterlogging stress^35^. Furthermore, application of exogenous GABA imparts partial protection from heat stress to rice seedlings by improving leaf turgor and up-regulating the expression of osmoprotectants and antioxidants^36^. Several other studies have indicated that the exogenous application of GABA promotes photochemical efficiency, chlorophyll biosynthesis, photosynthetic activities, enzymatic and non-enzymatic responses, and membrane stabilization in different crops^31,37,38^. Although endogenous GABA levels in plants are very low, it is produced rapidly by plants under stressful conditions^39^.

However, the adaptation of plants under abiotic stress depends on comprehensive responses based on physiological and biochemical changes, and the palliative effects of GABA on waterlogging stress still has not been fully elucidated. Therefore, in this study the exogenous application of GABA was initiated under waterlogging conditions at the third leaf stage (V3) and fifth leaf stage (V5), respectively, for two popular hybrid maize varieties in China: Zhengdan-958 (ZD-958) and Xing Ken-6 (XK-6). Plant growth responses and physiological adaptive mechanisms were evaluated; specifically, leaf gas exchange parameters, chlorophyll content, enzymatic and non-enzymatic systems, lipid peroxides and ROIs- accumulation, and chloroplast ultrastructure were examined. Our findings here will improve our understanding of the adaptation, survival, and tolerance of maize under waterlogging stress and will help to improve the growth status for higher yields under natural conditions.

## Results

### GABA application increased the GABA content in leaves under waterlogging stress

**Figure 1:**
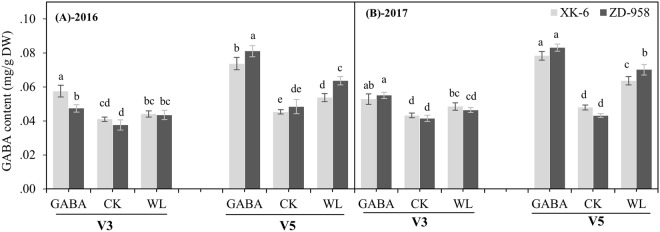
The GABA contents in leaves at 14 d after treatments initiated at third leaf (V3) and fifth leaf (V5) stage of maize under normal soil water conditions (CK), waterlogging treatment (WL) and aminobutyric acid treatment for waterlogged plants (GABA) in 2016 and 2017. Error bars represent the standard error (S.E.) of mean (n = 3). Different letters indicate significant differences at P < 0.05 based on the Least Significant Difference test.

As shown in Fig. 1, in comparison with CK treatment, GABA content in the leaves at V5 stage under the WL treatment was significantly increased, by 18.6% in XK-6 cultivar and 31.3% in ZD-958 cultivars in 2016 (*p* ≤ 0.05); however, slight increases in GABA content in the leaves at V3 stages under WL treatment were observed. Similar changes in GABA content were obtained between WL and CK treatments in 2017. Remarkably, exogenous application of GABA significantly increased GABA content in the leaves of maize seedlings under waterlogging stress at V3 and V5 stages (Fig. 1A,B). Compared with the WL and CK treatments, the GABA treatment significantly increased GABA content in the leaves at V3 stage, by 19.6% and 31.2% in XK-6, and by 13.7% and 29.3% in ZD-958, respectively, averaged across two experimental years. Moreover, improvement of GABA content was greater at V5 stages than V3 stages under GABA treatment in both maize varieties and years (Fig. 1A,B).

### GABA application enhances seedling growth under waterlogging stress

Results show that waterlogging for 14 d significantly (p ≤ 0.05) decreased plant height (PH), green leaf area (GLA), and shoot and root dry matter (DM) of two varieties, when compared with control plants (CK) in both years (Table 1). Noticeably, exogenous GABA application improved the performance in these growth parameters under waterlogging conditions compared with the WL plants (p ≤ 0.05), though they still showed weakness when compared with the CK plants. Among these growth parameters, root DM was increased by the largest extent for GABA application under waterlogging conditions, with increases of 57.6% at V3 stage and 74.4% at V5 stage, respectively, averaged across both varieties and years, when compared with the WL plants (Table 1). GABA treatment also showed a positive effect on aboveground DM of waterlogged plants for each variety at each treated growth stage, with an average increase of 19.0%. Green leaf area and plant height of maize seedlings under the GABA treatment was increased by 21.1% and 18.4%, relative to the WL treatment, averaged across both varieties, stages, and years, respectively, though no significant changes were observed at V3 stages in 2017.

**Table 1:** Effects of exogenous GABA application on growth attributes of XK-6 and ZD-958 maize seedlings under waterlogging stress in 2016 and 2017.

Varieties	Treatments	V3 stage				V5 stage			
		PH	GLA	Aboveground DM	Root DM	PH	GLA	Aboveground DM	Root DM
		(cm)	(cm^2^ plant^−1^)	(g plant^−1^)	(g plant^−1^)	(cm)	(cm^2^ plant^−1^)	(g plant^−1^)	(g plant^−1^)
**2016**									
XK-6	GABA	44.5 b	55.4 c	1.39 c	0.56 d	73.9 b	107.3 b	4.21 c	1.06 b
	CK	54.8 a	93.4 b	2.53 a	1.79 a	86.9 a	171.0 a	7.93 a	2.77 a
	WL	37.3 c	47.4 de	1.04 d	0.38 e	65.0 cd	91.1 c	3.50 d	0.72 c
ZD-958	GABA	39.4 c	51.7 cd	0.93 c	0.62 c	69.2 bc	99.1 bc	3.62 d	1.46 b
	CK	55.9 a	116.5 a	1.81 b	1.49 b	83.0 a	159.4 a	6.80 b	2.42 a
	WL	33.8 c	42.7 e	0.82 d	0.39 e	61.3 d	85.5 c	3.08 e	0.62 c
**ANOVA**									
Treatment	**	**	**	**	**	**	**	**	
Variety	ns	*	**	**	*	ns	**	ns	
Variety*Treatments	ns	**	**	**	ns	ns	*	ns	
**2017**									
XK-6	GABA	56.7 b	77.7 bc	1.94 b	0.74 c	72.3 c	112.6 d	5.75 d	1.49 b
	CK	75.6 a	128.8 a	4.69 a	2.00 a	98.3 a	179.7 b	9.15 b	2.87 a
	WL	45.6 c	63.4 c	1.56 c	0.48 d	64.3 c	85.5 f	4.98 f	1.02 c
ZD-958	GABA	58.6 b	83.3 b	1.92 b	0.93 b	87.0 b	122.4 c	6.20 c	1.59 b
	CK	79.2 a	120.9 a	4.85 a	1.90 a	93.7 ab	196.1 a	9.38 a	2.70 a
	WL	50.2 bc	67.0 bc	1.57 c	0.45 d	66.4 c	103.3 e	5.22 e	0.95 c
**ANOVA**									
Treatment		**	**	**	**	**	**	**	**
Variety		ns	*	ns	*	*	ns	**	ns
Variety*Treatments		ns	**	ns	ns	ns	ns	ns	ns

** and *denote significance at the 0.01 and 0.05 probability level, respectively; ns, non-significant. Values followed by a different small letter within a column are significantly different at 5% probability level for comparison among varieties under different treatments at same growth stage in the same year. GABA, treatment of GABA application to waterlogged maize seedlings; WL, waterlogging treatment; CK, normal soil water conditions; PH, plant height; GLA, green leaf area; Above ground DM, shoot and leaf dry matter; root DM, root dry matter.

### GABA application improves photosynthesis under waterlogging stress

**Figure 2:**
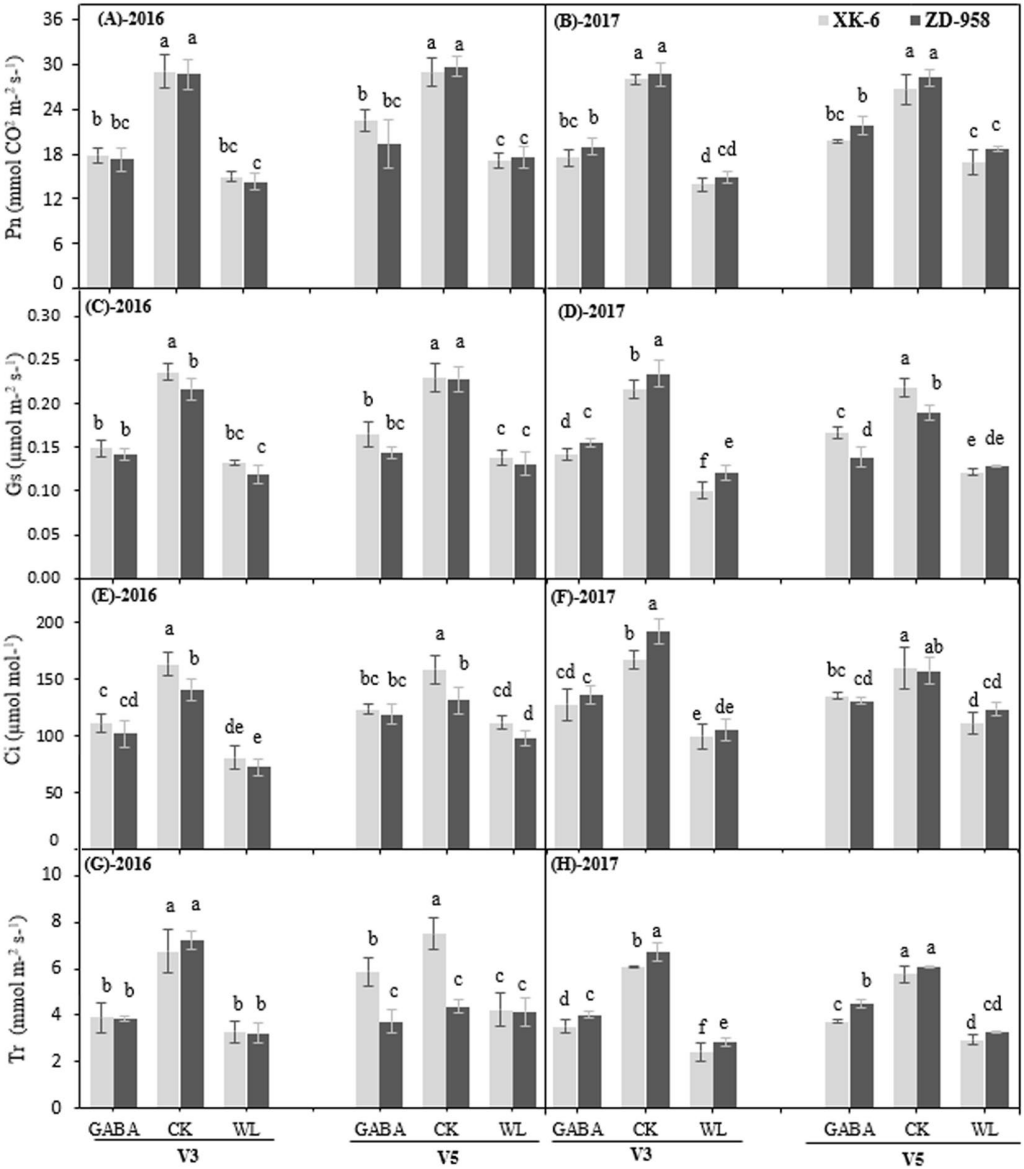
Effect of exogenous GABA application on net photosynthetic rate (Pn), (**A**,**B**) stomatal conductance (Gs), (**C**,**D**) intercellular CO_2_ concentration (Ci), (**E**,**F**) and transpiration rate (Tr), (G and H) in leaves at 14 d after treatment initiated at third leaf (V3) and fifth leaf (V5) stage of maize in 2016 and 2017. Samples were analyzed under normal soil water conditions (CK), waterlogging treatment (WL) and aminobutyric acid treatment for waterlogged plants (GABA). Error bars represent the standard error (S.E.) of mean (n = 3). Different letters indicate significant differences at P < 0.05 based on the Least Significant Difference test.

Leaf gas exchange parameters of maize seedlings were significantly decreased by waterlogging stress, when compared with the control plants at V3 and V5 stages (Fig. 2). Under waterlogging, average net photosynthetic rate (P_n_), stomatal conductance (G_s_), intercellular CO_2_ (C_i_), and transpiration rate (T_r_) of maize seedlings were decreased by 49%, 48%, 46%, and 56% in XK-6 and 42%, 38%, 35%, and 51% in ZD-958, respectively, when compared with that under CK **(**Fig. 2**)**. The plants treated with exogenous GABA application improved P_n_, G_s_, C_i_, and T_r_ by 19.8%, 19.4%, 21.5%, 26.2%, respectively, compared with WL treatments averaged across two varieties, stages, and years. However, there was no significant increase in GABA application on photosynthetic rate (P_n_) and transpiration rate (T_r_) were observed in 2016 years compared with WL treatment. Minimal differences were observed in leaf gas exchange parameters between two varieties under the same treatment.

### GABA application decreases lipid peroxidation and ROIs-producing enzymes under waterlogging stress

**Figure 3:**
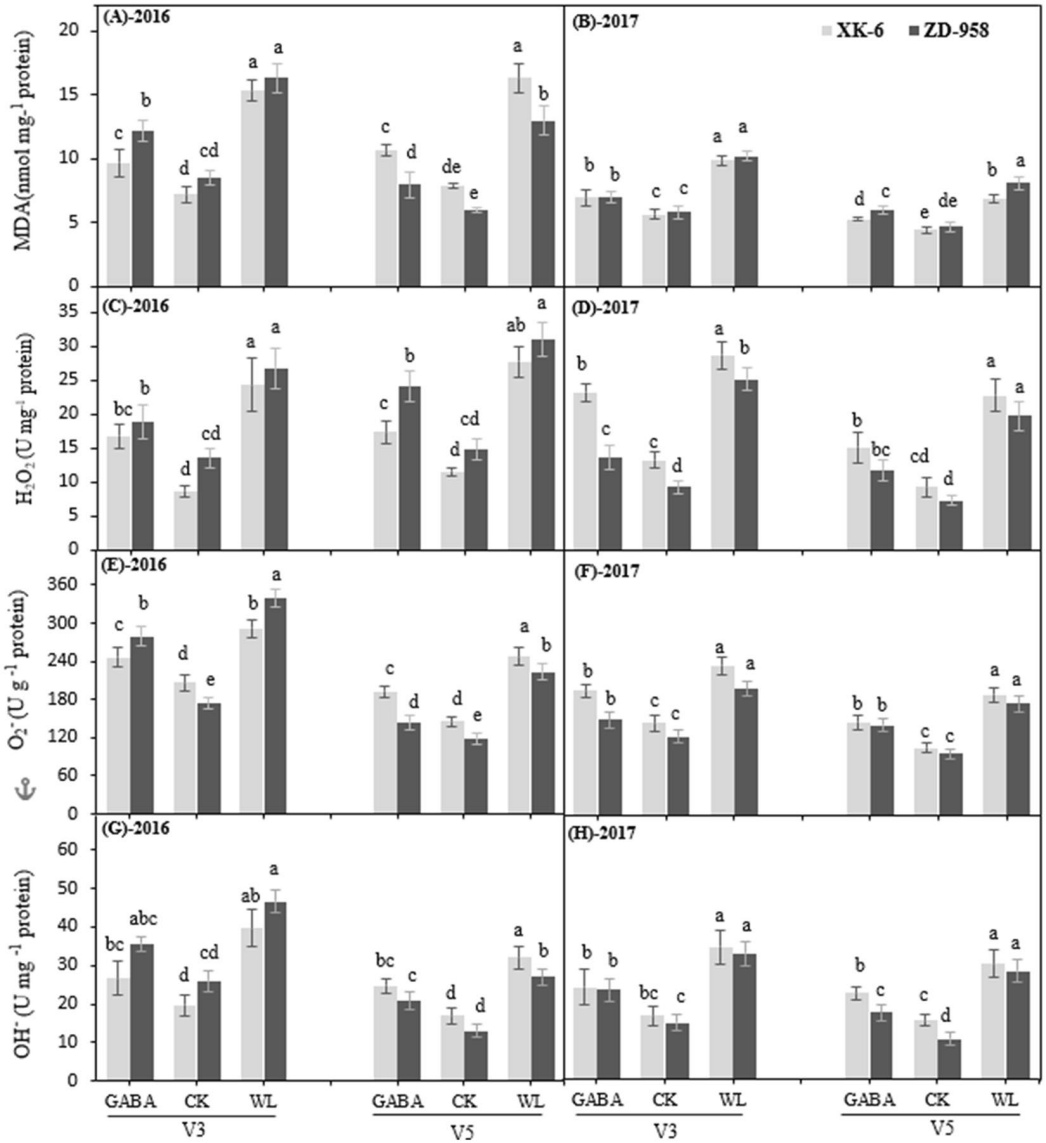
Determination of changes in malondialdehyde (MDA) content (**A**,**B**), hydrogen peroxide (H_2_O_2_) content (**C**,**D**), superoxide anion radical production (O_2_˙^−^) content (**E**,**F**) and hydroxyl ion (OH^−^) content (**G**,**H**) in leaves at 14 d after treatment initiated at third leaf (V3) and fifth leaf (V5) stage of maize in 2016 and 2017. Samples were analyzed under normal soil water conditions (CK), waterlogging treatment (WL) and aminobutyric acid treatment for waterlogged plants (GABA). Error bars represent the standard error (S.E.) of mean (n = 3). Different letters indicate significant differences at P < 0.05 based on the Least Significant Difference test.

Waterlogging stress triggered the lipid peroxidation and production of ROIs; thus, accumulation of Malondialdehyde (MDA), hydrogen peroxide (H_2_O_2_), ^⋅^O_2_^−^, and ^⋅^OH^−^ were remarkably increased in waterlogged maize seedlings (Fig. 3). The values of MDA, H_2_O_2_, ^⋅^O_2_^−^, and ^⋅^OH^−^ content in maize leaves were obviously alleviated under GABA treatment by 30.5%, 32.5%, 21.8%, and 21.0%, averaged across different varieties, stages, and years, respectively, compared with the WL treatment (Fig. 3). It appears that GABA application had a more positive effect on reducing MDA and H_2_O_2_ accumulation than on ^⋅^O_2_^−^ and ^⋅^OH^−^.

**Figure 4:**
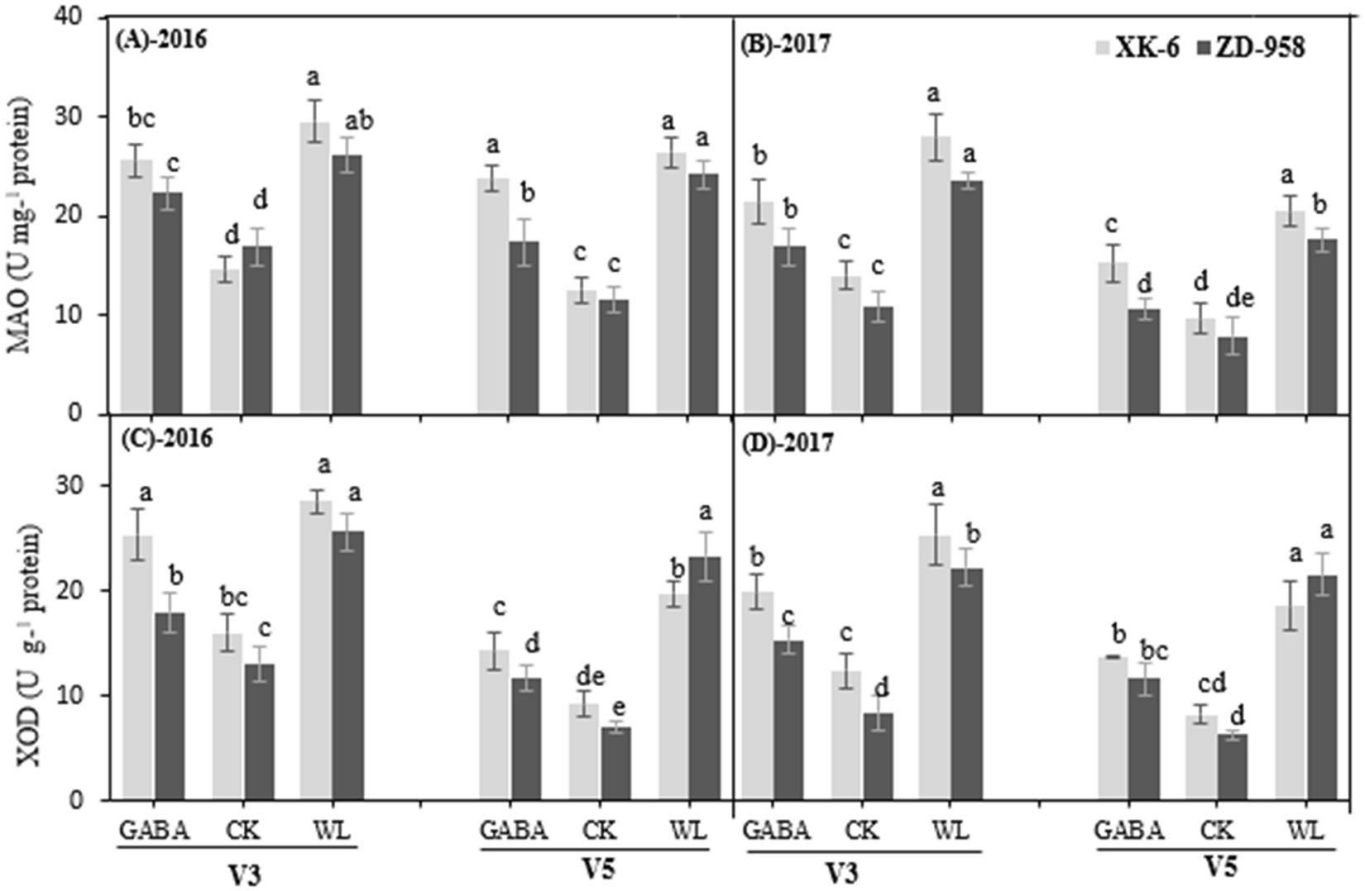
Activities of monoamine oxidase (**A**,**B**) and Xanthine Oxidase (**C**,**D**) in leaves at 14 d after treatment initiated at third leaf (V3) and fifth leaf (V5) stage of maize in 2016 and 2017. Samples were analyzed under normal soil water conditions (CK), waterlogging treatment (WL) and aminobutyric acid treatment for waterlogged plants (GABA). Error bars represent the standard error (S.E.) of mean (n = 3). Different letters indicate significant differences at P < 0.05 based on the Least Significant Difference test.

In contrast, the activities of ROIs-producing enzymes, monoamine oxidase and xanthine oxidase (MAO and XOD), in the leaves were significantly higher under WL treatment and significantly reduced in GABA application treatments in most cases (Fig. 4). In plants treated with exogenous GABA, the activity of MAO and XOD were restrained by 18.0% and 21.6% in XK-6 and 27.6% and 39.1% in ZD-958, respectively, compared with that under the WL stressed plants averaged across two stages and years **(**Fig. 4). GABA application had a more profound effect on MAO than on XOD, and on ZD-958 than on XK-6. However, no significant changes in MAO at the V5 stage in 2016 and in XOD at the V3 stage in 2017 were found between XK- 6 plants under GABA and WL treatments (Fig. 4A,C).

### GABA application fortifies antioxidants in cells under waterlogging stress

**Figure 5:**
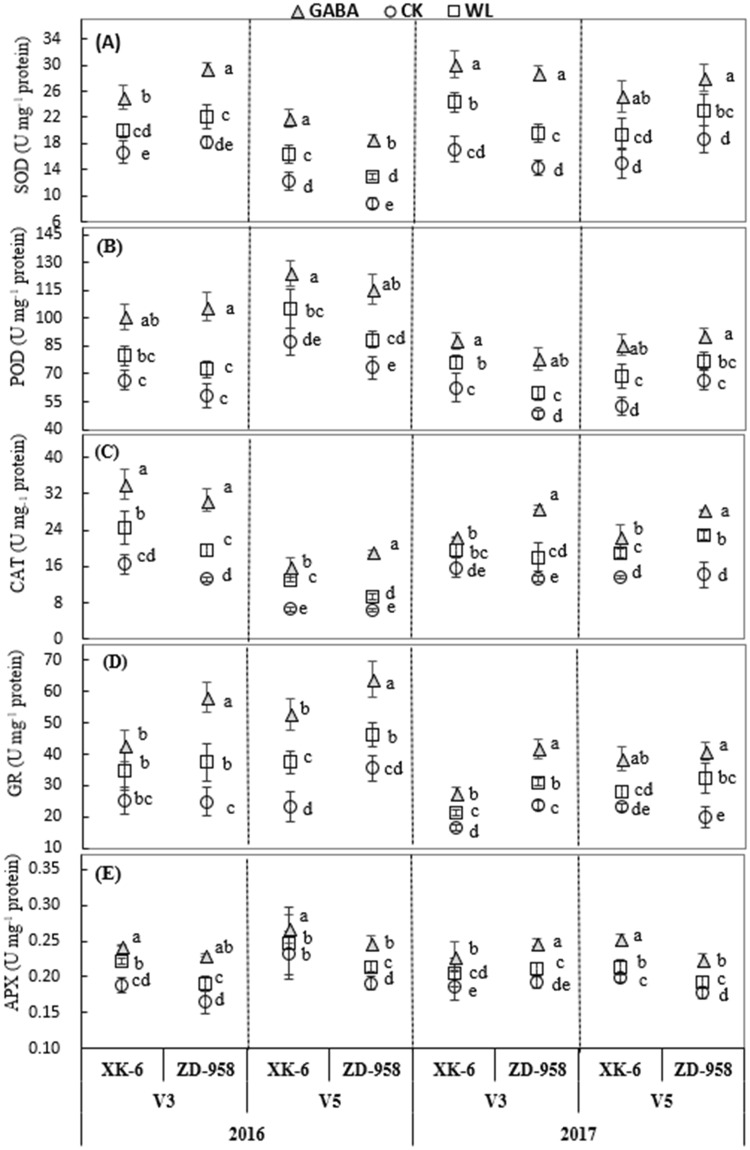
The activities of superoxide dismutase (SOD) (**A**), peroxidase (POD), (**B**), catalase (CAT), (**C**), ascorbate peroxidase (APX) (**D**) and glutathione reductase (GR) (**E**) in leaves at 14 d after treatment initiated at third leaf (V3) and fifth leaf (V5) stage of maize in 2016 and 2017. Samples were analyzed under normal soil water conditions (CK), waterlogging treatment (WL) and aminobutyric acid treatment for waterlogged plants (GABA). Error bars represent the standard error (S.E.) of mean (n = 3). Different letters indicate significant differences at P < 0.05 based on the Least Significant Difference test.

Although antioxidant enzyme activities in leaves had apparent variations across different maize growth stages and experimental years, their values were significantly increased under GABA application and WL treatment (p ≤ 0.05) compared with the control under the same stage in the same year (Fig. 5). Moreover, the activities of superoxide dismutase (SOD), peroxidase (POD), catalase (CAT), ascorbate peroxidase (APX) and glutathione reductase (GR), were significantly (p ≤ 0.05) increased under GABA application, when compared with the same maize variety under WL treatment at both V3 and V5 stages. The increase in CAT under GABA application was particularly high, with an increase of 42.7% averaged across varieties, stages, and years (Fig. 5C). Exogenous GABA application enhanced SOD and GR activities, with increases of 32.2% and 35.5%, respectively, averaged across varieties, stages, and years. A relatively small increase in APX was found under GABA treatment (14.7%); meanwhile, increases in APX were similar among different varieties and growth stages in both years (Fig. 5E). As shown in Fig. 4A–D, the ZD-958 variety under GABA treatment showed a higher increase in SOD, POD, CAT, and GR at the V3 stage (40.0%, 38.8%, 58.1%, and 45.0%, respectively), while the XK-6 variety under GABA treatment only showed increases of 25.0%, 20.7%, 26.8%, and 25.8% at the same stage, respectively. At the V5 stage, increases in SOD, POD, and GR were similar between varieties under GABA application; however, increases in CAT activity in ZD-958 leaves under GABA treatment was higher than that in XK-6 leaves compared with the same variety under WL treatment (Fig. 5C).

**Figure 6:**
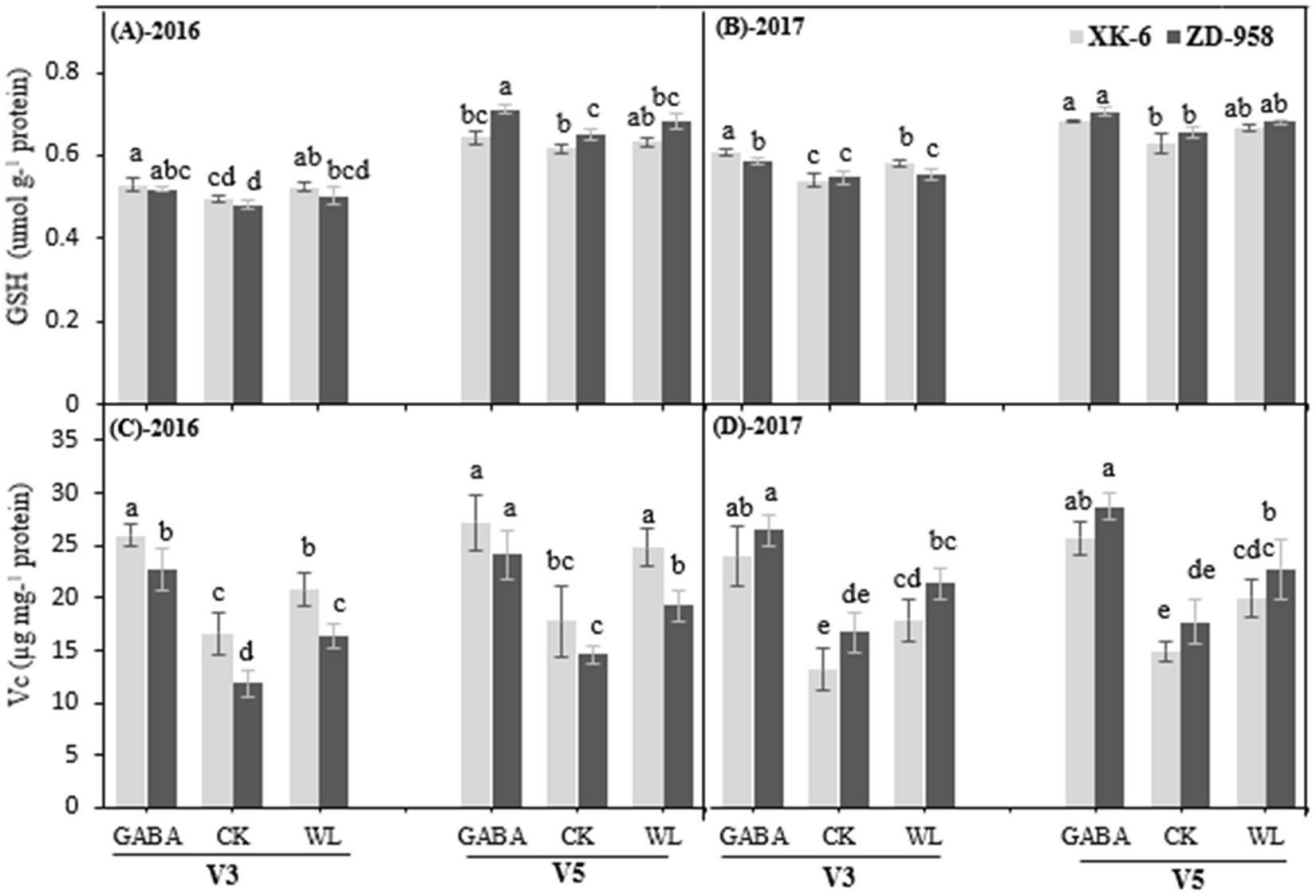
Effects of spraying GABA after waterlogging on non-enzymatic antioxidants in leaves at 14 d after treatment initiated at third leaf (V3) and fifth leaf (V5) stage of maize in 2016 and 2017. GSH (**A**,**B**) and V_C_ (**C**,**D**) Samples were analyzed under normal soil water conditions (CK), waterlogging treatment (WL) and aminobutyric acid treatment for waterlogged plants (GABA). Error bars represent the standard error (S.E.) of mean (n = 3). Different letters indicate significant differences at P < 0.05 based on the Least Significant Difference test.

The GSH content did not show consistently significant increases in the WL treated maize seedlings, when compared with the CK plants (Fig. 6A,B). At V3 and V5 stages, the GSH content in plants treated with exogenous GABA was slightly increased, by 2.8% and 2.4% in XK-6 and by 4.5% and 3.9% in ZD-958, respectively, when compared with waterlogging-stressed plants. Nevertheless, the VC content were significantly enhanced in both growth stages of two varieties under waterlogging conditions with GABA application, when compared with plants under CK or WL treatments (Fig. 6C,D). After spraying exogenous GABA, the VC content increased by 29% and 31% in XK-6 and ZD-958 at the V3 stage and 19% and 26% at V5 stage, respectively, compared with the WL treatment.

### GABA application increases chlorophyll content and chloroplasts under waterlogging stress

Chlorophyll content of maize leaves was significantly affected (*p* ≤ 0.05) at V3 and V5 stages by waterlogging stress compared with CK plants (Table 2). WL treatment significantly decreased chlorophyll content, with reductions of 44% and 44% at the V3 stage for XK-6 and ZD-958, respectively, and 41% and 52% at V5 stage for XK-6 and ZD-958, respectively, averaged across years, when compared with CK. By contrast, chlorophyll content was significantly increased in the GABA treatments, by 32% and 43%, at the V5 stage and 32% and 37% at the V3 stage (p ≤ 0.05) for XK-6 and ZD-958, respectively, when compared with the WL treatment (Table 2). Chloroplast numbers per mesophyll cell in leaves of two varieties were significantly reduced (p ≤ 0.05) under WL conditions at two stages in two years (Table 2). At the V5 stage, the GABA and WL treatments exhibited reduced number of chloroplasts, by 26% and 42% in XK-6 and by 31% and 52% in ZD-958, respectively, averaged across years. However, the plants treated with exogenous GABA application under waterlogging stress exhibited obvious increases number of chloroplast at both stages of two varieties in 2016 in compared with WL treatments, though these increases were not observed in 2017 year.

**Table 2:** Effects of exogenous GABA on chloroplast ultrastructure characteristic of mesophyll cell in maize seedling of XK-6 and Zhangdan958 under waterlogging stress during growing period 2016 and 2017.

Stages	Varieties	Treatments	Chl content (mg g^−1^ FW)	Chloroplast number per mesophyll cell	Grana number per chloroplast	Chloroplast size	
						Length (µm)	Width(µm)
**2016**							
V3	XK-6	GABA	1.26 cd	10.0 ef	22.5 de	7.02 defg	3.43 ab
		CK	1.97 b	11.7 cde	28.2 a	8.66 b	3.59 ab
		WL	0.70 h	7.0 g	14.8 fg	5.33 h	2.75 bcde
	ZH-958	GABA	1.05 ef	10.7 b	18.0 ef	6.03 fgh	3.22 bc
		CK	1.96 b	14.7 b	26.2 ab	8.53 bc	4.46 a
		WL	0.79 gh	8.0 fg	13.3 g	7.16 cdef	2.74 bcde
V5	XK-6	GABA	1.39 c	10.7 def	22.8 bcd	6.36 efgh	2.28 cdef
		CK	2.19 a	14.3 bc	29.5 a	7.31bcde	2.84 bcd
		WL	0.95 fg	6.7 g	15.3 fg	5.67 gh	1.61 f
	ZH-958	GABA	1.15 de	13.3 bcd	24.5 bc	8.23 bcd	2.04 def
		CK	2.08 ab	20.0 a	29.5 a	11.20 a	3.05 bcd
		WL	0.92 fg	9.0 efg	20.5 de	7.71 bcde	1.69 ef
**ANOVA**							
Variety		*	**	ns	**	ns	
Treatment		**	**	**	**	*	
Stage		ns	ns	**	ns	**	
Stage*Treatments		ns	ns	ns	*	ns	
Variety*Treatments		*	ns	ns	ns	ns	
**2017**							
V3	XK-6	GABA	1.18b	7.0 de	18.5 ef	6.22 bcd	2.71 bcde
		CK	1.40 cd	11.0 b	30.3 b	7.90 a	3.39 b
		WL	0.96 g	5.8 e	13.5 g	4.72 e	1.16 g
	ZH-958	GABA	1.22 ef	7.0 de	23.0 cd	5.37 cde	1.70 efg
		CK	1.33 de	8.7 cd	33.0 b	6.29 bcd	2.96 bcd
		WL	0.92 g	5.0 e	19.0 ef	4.36 e	1.84 defg
V5	XK-6	GABA	1.39 cd	8.3 cd	20.3 de	5.04 de	1.96 cdefg
		CK	1.65 b	11.3 b	31.0 b	7.44 ab	3.12 bc
		WL	1.23 ef	8.0 cd	16.3 fg	5.23 de	1.83 defg
	ZH-958	GABA	1.50 c	9.7 bc	26.0 c	6.55 bc	2.34 bcdef
		CK	1.77 a	13.7 a	37.0 a	8.57 a	4.78 a
		WL	1.26 ef	7.0 de	17.8 ef	4.91 e	1.41 fg
**ANOVA**							
Variety			ns	ns	**	ns	ns
Treatment			**	**	**	*	*
Stage			*	*	ns	ns	ns
Stage*Treatments			ns	ns	ns	ns	ns
Variety*Treatments			ns	ns	ns	ns	ns

** and *denote significance at the 0.01 and 0.05 probability level, respectively; ns, non-significant. Values followed by a different small letter within a column are significantly different at 5% probability level for comparison among varieties under different treatments at same growth stage in the same year. GABA, treatment of GABA application to waterlogged maize seedlings; WL, waterlogging treatment; CK, normal soil water conditions.

### GABA application improved chloroplast ultrastructure in mesophyll cells and mitochondrion structure

**Figure 7:**
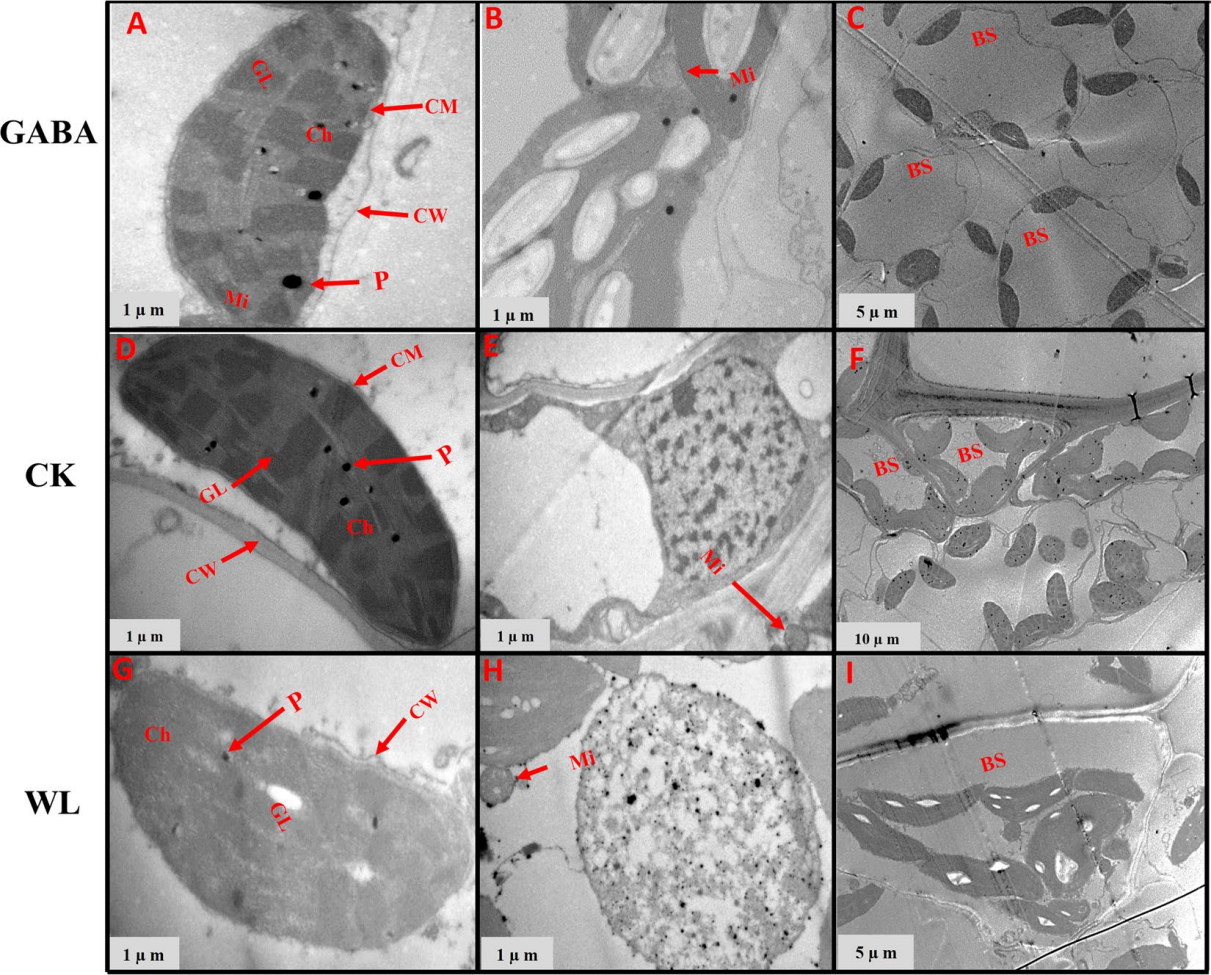
Chloroplast ultrastructure in leaves of XK-6 maize cultivars under different treatments. Chloroplast ultrastructure under GABA application for waterlogging maize seedlings (GABA) at 14 d after treatment initiated at third leaf (V3) in 2016. Ch: chloroplast, GL: grana lamella, P: particles, CM: chloroplast envelope membrane, CW: cell wall, Mi: mitochondria, BS: bundle sheath.

**Figure 8:**
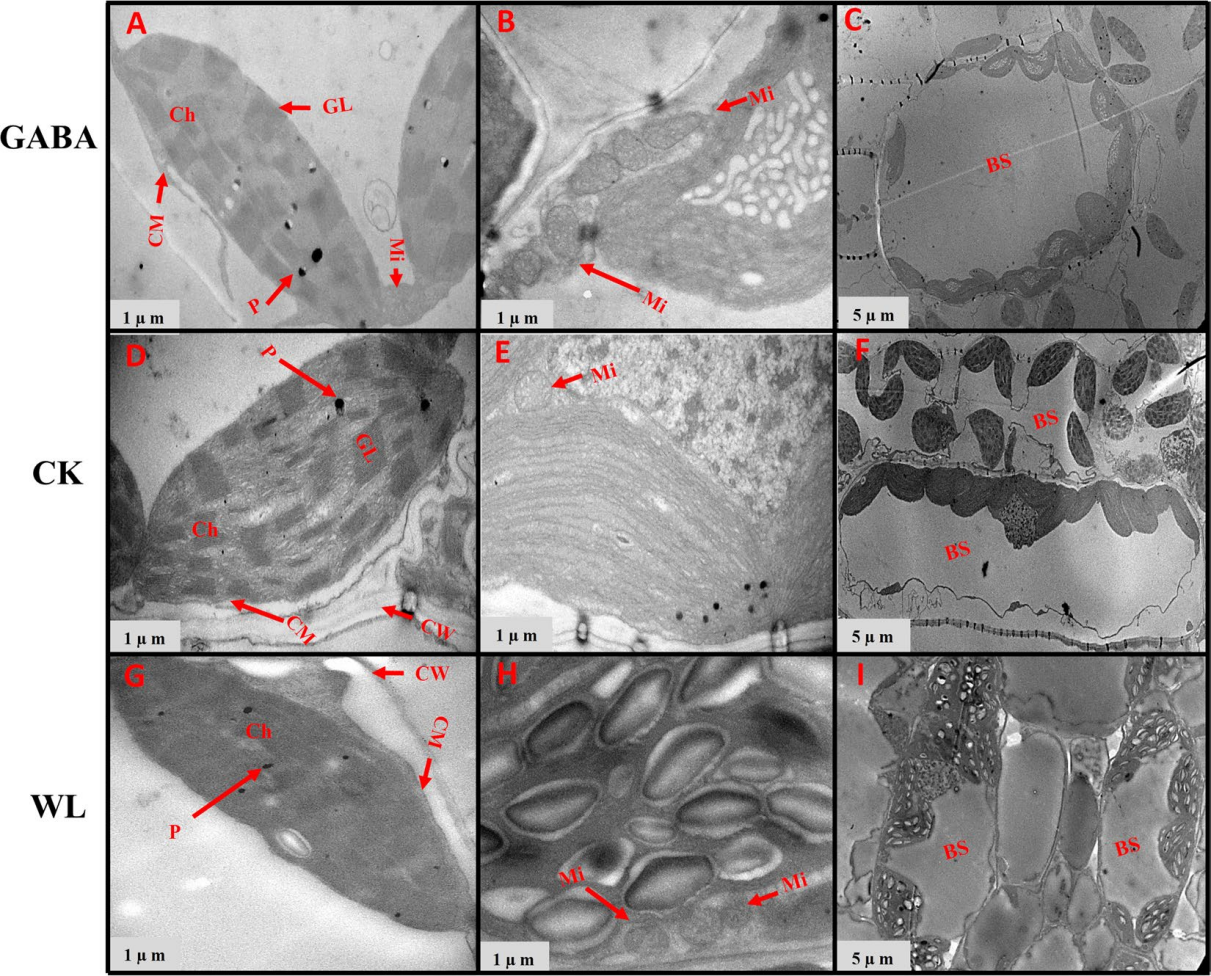
Chloroplast ultrastructure in leaves of ZD-958 maize cultivars under different treatments. Chloroplast ultrastructure under GABA application for waterlogging maize seedlings (GABA) at 14 d after treatment initiated at third leaf (V3) in 2016. Ch: chloroplast, GL: grana lamella, P: particles, CM: chloroplast envelope membrane, CW: cell wall, Mi: mitochondria, BS: bundle sheath.

**Figure 9:**
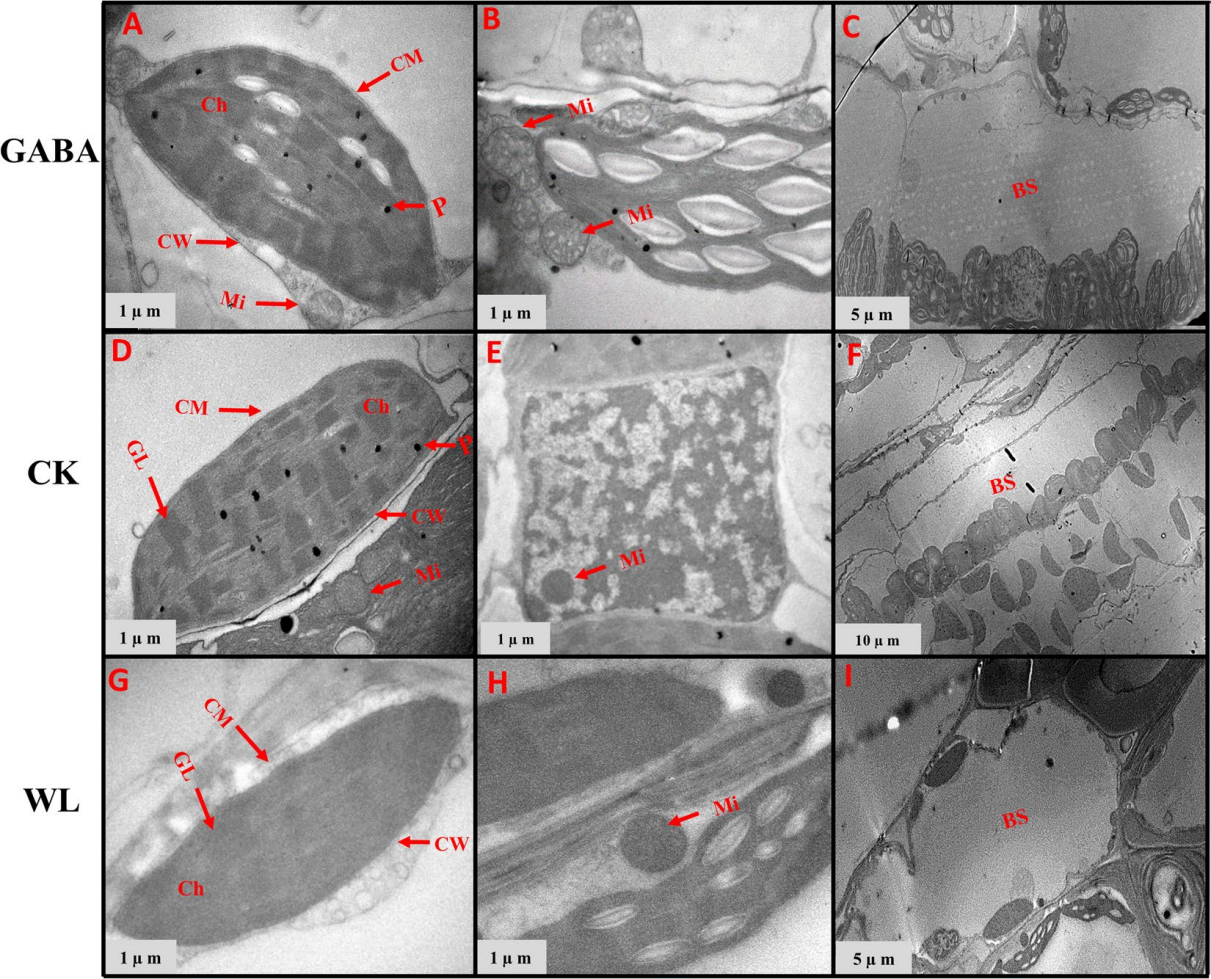
Chloroplast ultrastructure in leaves of XK-6 maize cultivars under different treatments. Chloroplast ultrastructure under GABA application for waterlogging maize seedlings (GABA) at 14 d after treatment initiated at five leaf (V5) stage in 2016. Ch: chloroplast, GL: grana lamella, P: particles, CM: chloroplast envelope membrane, CW: cell wall, Mi: mitochondria, BS: bundle sheath.

**Figure 10:**
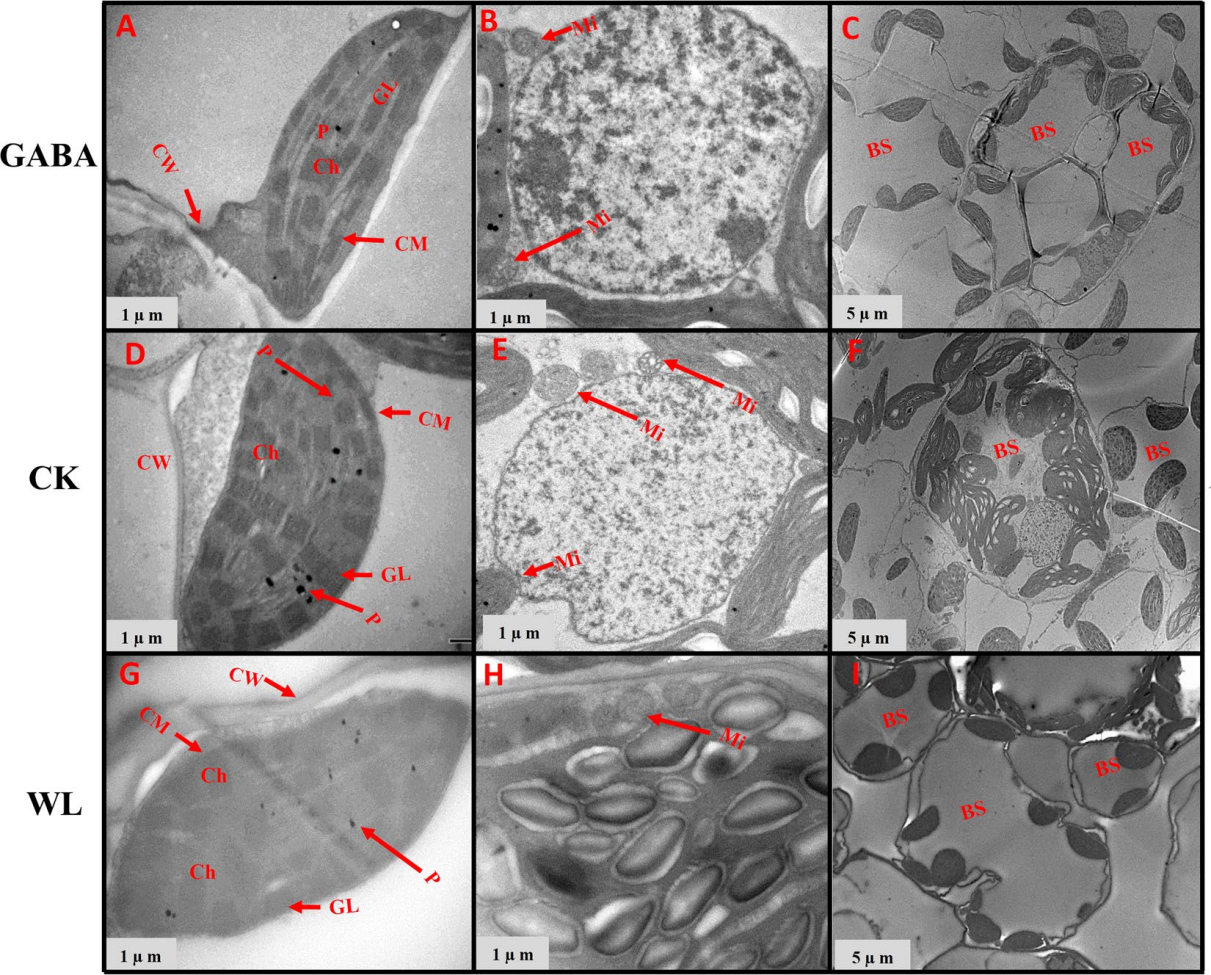
Chloroplast ultrastructure in leaves of ZD-958 maize cultivars under different treatments. Chloroplast ultrastructure under GABA application for waterlogging maize seedlings (GABA) at 14 d after treatment initiated at five leaf (V5) stage in 2016. Ch: chloroplast, GL: grana lamella, P: particles, CM: chloroplast envelope membrane, CW: cell wall, Mi: mitochondria, BS: bundle sheath.

**Figure 11:**
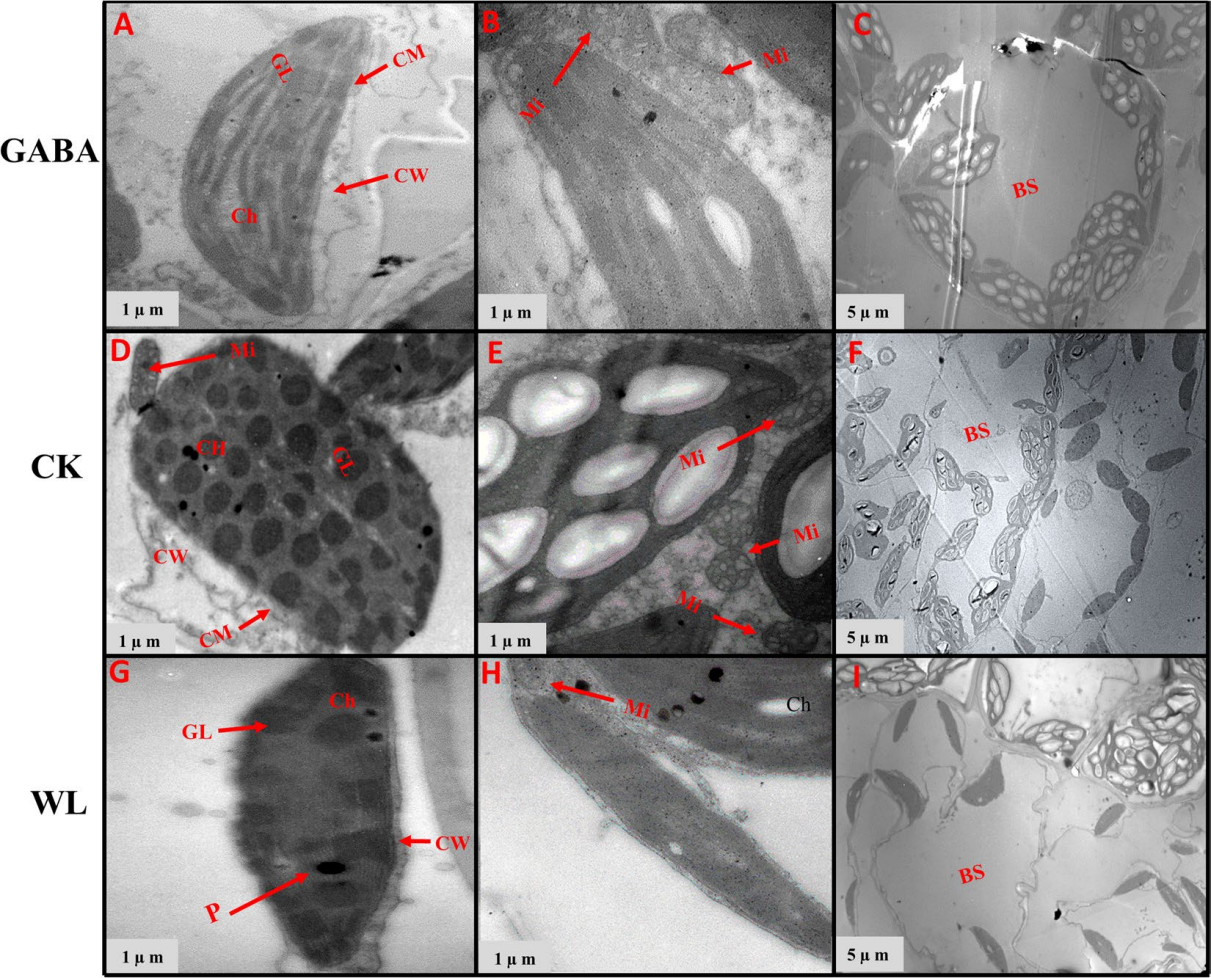
Chloroplast ultrastructure in leaves of XK-6 maize cultivars under different treatments. Chloroplast ultrastructure under GABA application for waterlogging maize seedlings (GABA) at 14 d after treatment initiated at third leaf (V3) in 2017. Ch: chloroplast, GL: grana lamella, P: particles, CM: chloroplast envelope membrane, CW: cell wall, Mi: mitochondria, BS: bundle sheath.

**Figure 12:**
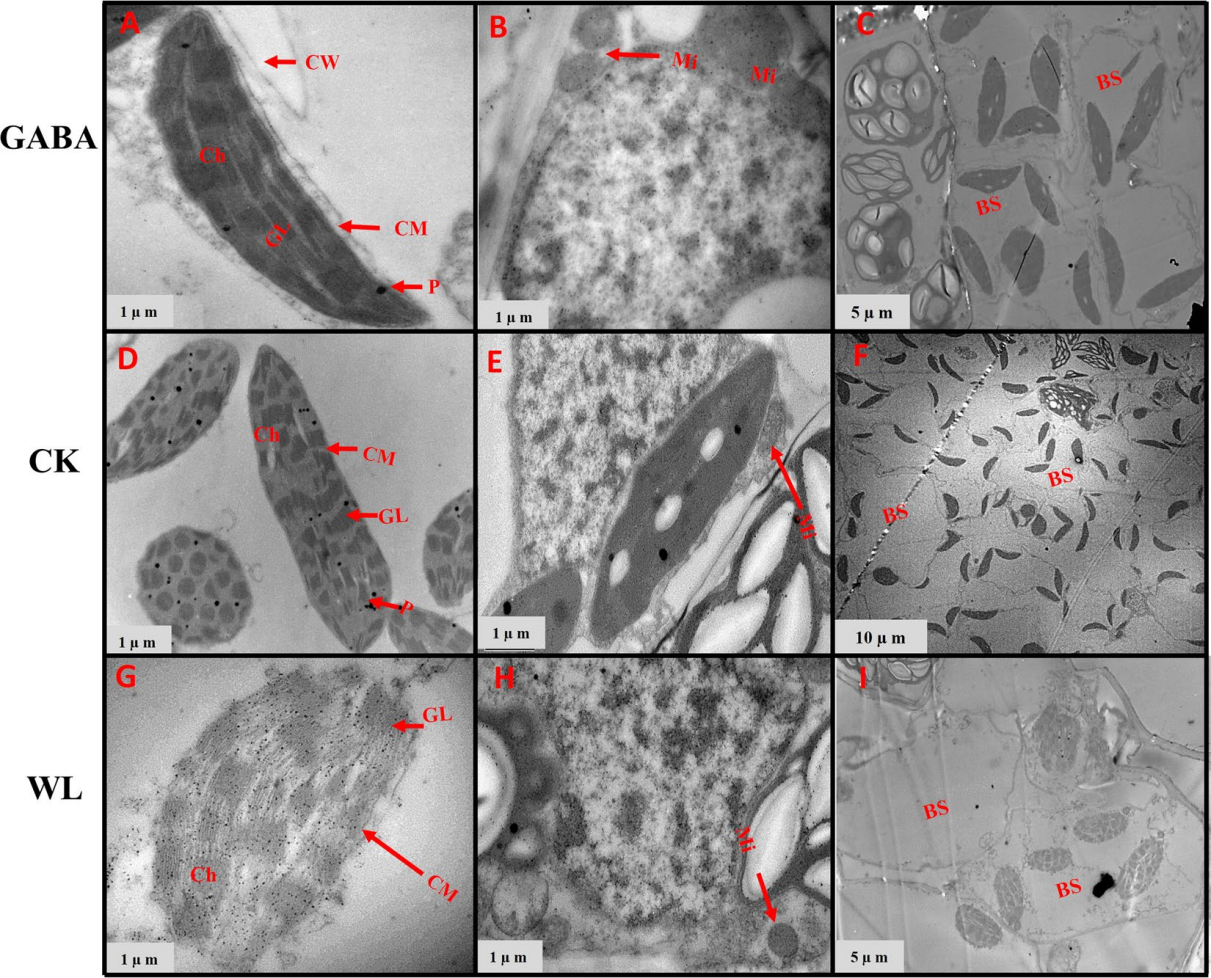
Chloroplast ultrastructure in leaves of ZD-958 maize cultivars under different treatments. Chloroplast ultrastructure under GABA application for waterlogging maize seedling (GABA) at 14 d after treatment initiated at third leaf (V3) in 2017. Ch: chloroplast, GL: grana lamella, P: particles, CM: chloroplast envelope membrane, CW: cell wall, Mi: mitochondria, BS: bundle sheath.

**Figure 13:**
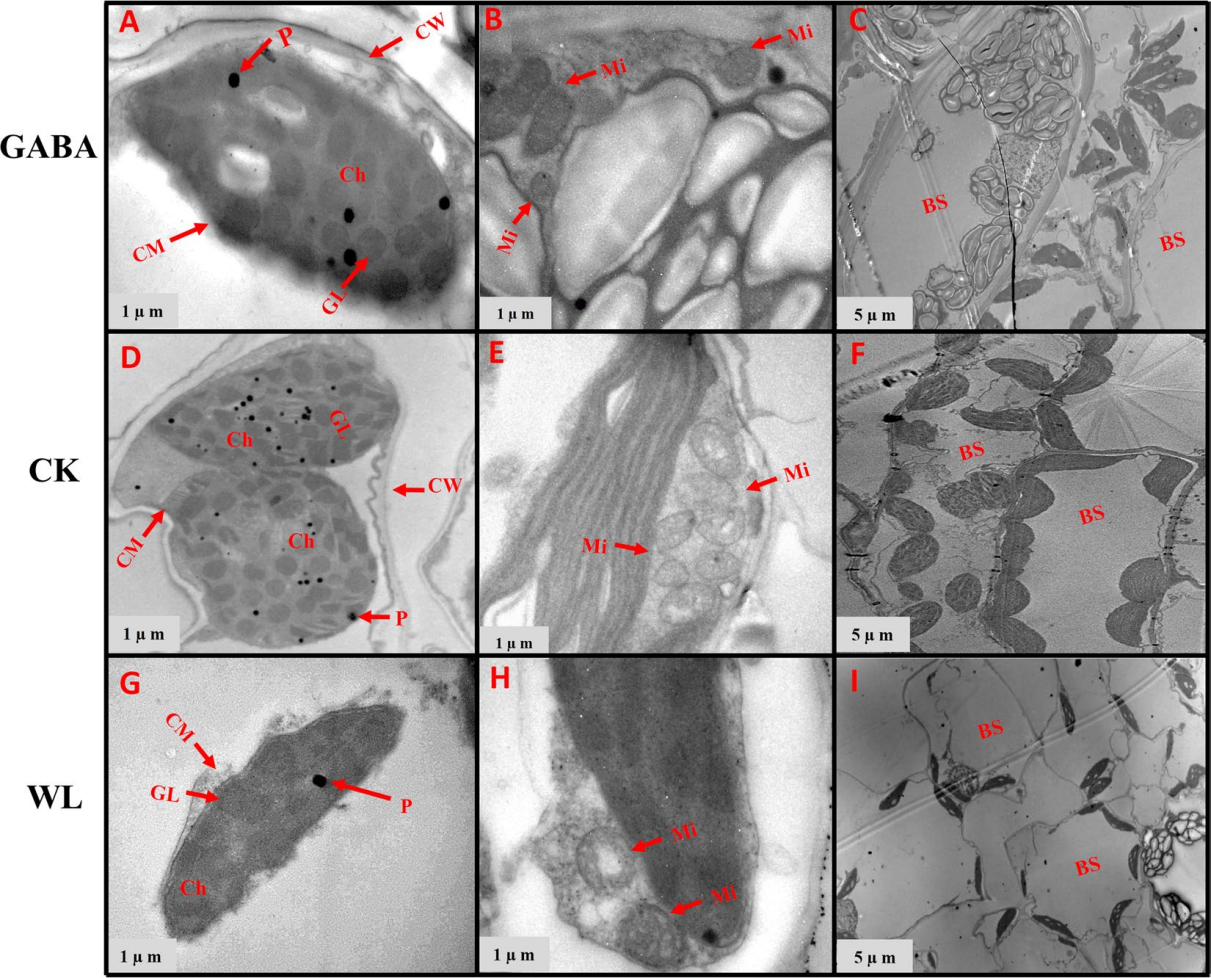
Chloroplast ultrastructure in leaves of XK-6 maize cultivars under different treatments. Chloroplast ultrastructure under GABA application for waterlogging maize seedling (GABA) at 14 d after treatment initiated at five leaf (V5) stage in 2017. Ch: chloroplast, GL: grana lamella, P: particles, CM: chloroplast envelope membrane, CW: cell wall, Mi: mitochondria, BS: bundle sheath.

**Figure 14:**
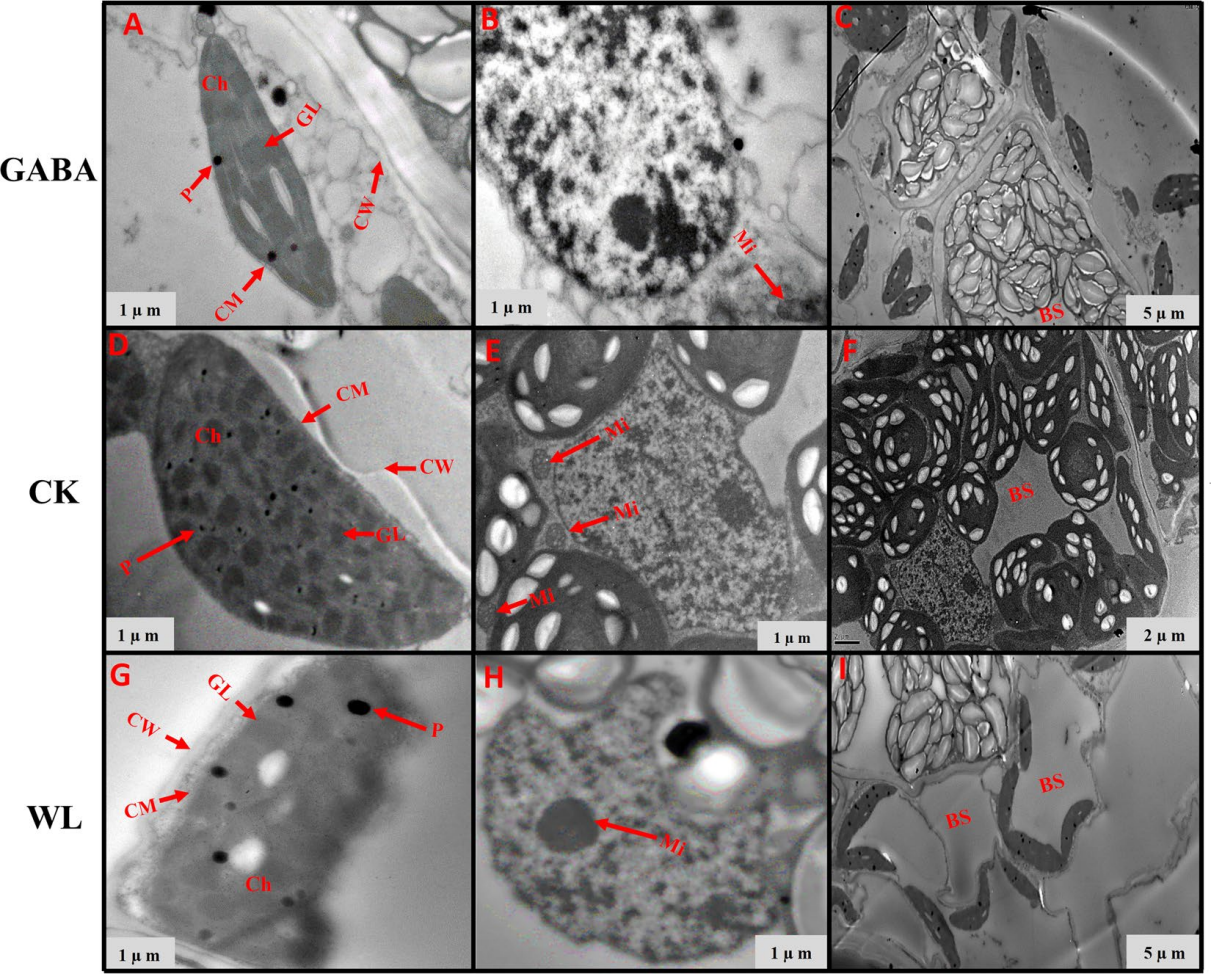
Chloroplast ultrastructure in leaves of ZD-958 maize cultivars under different treatments. Chloroplast ultrastructure under GABA application for waterlogging maize seeding’s (GABA) at 14 d after treatment initiated at five leaf (V5) stage in 2017. Ch: chloroplast, GL: grana lamella, P: particles, CM: chloroplast envelope membrane, CW: cell wall, Mi: mitochondria, BS: bundle sheath.

In control plants, the chloroplasts had complete external envelopes and clear boundaries, the thylakoid were well-developed with good shape, the lamella structure pile folds were in order, and both grana lamella and stroma lamellae were arranged compactly and clearly in both maize cultivars (Figs 7D–14D). However, chloroplast configuration became irregular under WL conditions. Chloroplasts in maize seedlings under waterlogging stress were characterized by compressed grana lamellae, disrupted stroma lamellae, and distorted thylakoid. Additionally, the number of grana and grana lamellae were reduced significantly to varying degrees (Figs 7G–10G and 12G–14G). At the V3 stage, the number of grana was reduced by 49.8% and 43.7% in XK-6 and ZD-958 plants under WL treatment, respectively, compared with CK (Table 2, Figs 7 and 8). However, the plants treated with GABA application had a consistently positive effect on number of grana of maize varieties under waterlogging stress in both years, and significantly increased the number of grana per chloroplast by an average of 41% and 31% in XK-6 and ZH-958, respectively, relative to WL treatment (Table 2). The size of chloroplasts in maize seedlings waterlogged under WL treatment and GABA treatment were obviously reduced compared with those in CK plants (Table 2, Figs 7, 8 and 14). In comparison with CK plants, the length and width of chloroplasts under WL treatments were significantly decreased by 33% and 43% in XK-6 and 30% and 48% in ZD-958, averaged across stages and years (Table 2). However, application of GABA to waterlogged maize seedlings did not result in increased chloroplast size in most experimental conditions (Table 2). In contrast with the WL treatment, significant increases in the length of chloroplasts by GABA treatment were only found in XK-6 plants at the V3 stage in 2016 and in ZD-958 plants at the V5 stage in 2017 (Table 2). Additionally, only maize seedlings at the V3 stage of XK-6 in 2017 increased in chloroplast width under GABA treatment (Table 2). Nevertheless, exogenous GABA alleviated the ultrastructure changes of chloroplasts in mesophyll cells induced by waterlogging **(**Figs 7–9, 12 and 14). The plants treated with GABA maintained a well-protected internal lamellar system in the chloroplasts of waterlog-stressed leaves in both maize seedlings **(**Figs 7A–9A, 12A and 14A).

In control leaf cells, mitochondria were arranged randomly in the cytoplasm and were almost elliptical or circular. The double membrane structure was complete and crests were clearly visible (Figs 7E–10E, 12E and 14E). However, waterlogging stress altered the structure of mitochondria to certain extent. Mitochondria in the WL treatment were in clustered arrangements near the chloroplasts and were not clearly visible. (Figs 7H–9H, 12H and 14H). In contrast, there was no effect on mitochondria structure in the GABA treatment when compared with the WL treatment (Figs 11E and 14E). However, GABA alleviated the waterlogging-induced damage of mitochondria structure. An increased number of mitochondria and improved mitochondria structure were observed in both growth stages **(**Figs 7B–10B,12B and 14B).

## Discussion

GABA is the main component of the free ammonia acid library in plant cells. GABA usually accumulates in plants experiencing environmental stress, such as oxygen deficit, chilling, heat stimulus, or mechanical damage^39,40^. Similarly, increases in GABA content in maize seedling leaves under waterlogging stress were observed in this study (Fig. 1). GABA is a non-protein four-carbon amino acid that can be absorbed directly by plants^25–28^. In the present study, exogenous application of GABA significantly enhanced GABA content in maize seedlings under waterlogging conditions at V3 and V5 stages compared with CK and WL treatments (Fig. 1A,B). Such absorption of exogenous GABA by plants has also been reported in previous studies^41,42^. Moreover, we found that maize seedlings at the V5 stage had a stronger ability to produce GABA and absorb exogenous GABA than the plants at V3 stages under waterlogging stress (Fig. 1A,B); this might be one of the reasons that maize plants at the V5 stage had a greater ability to tolerate waterlogging stress, shown by better growth attributes in these plants (Table 1).

It has been reported that GABA can ameliorate plant growth under different abiotic stress conditions in maize, rice, brassica, and wheat^32,36,38–43^. However, GABA’s role in plants has remained unclear, while its role as a neurotransmitter in animal cells is well recognized. The major roles of GABA are regulating cytosolic pH, anti-oxidative enzymatic systems, buffering C and N metabolism, osmoregulation, armoring against oxidative stress, and signal transduction, and these might lead to improvement in overall plant performance^44,45^. The effect of GABA application to maize seedlings under waterlogging stress was examined in the present study, and it was found that waterlogging stress had profound adverse effects on maize seedling growth (Table 1), similar to reports in previous studies^3,14,46,47^. However, exogenous GABA application improved these plant growth attributes when plants were subjected to waterlogging stress (Table 1). Exogenous GABA application on leaves significantly increased the plant height and leaf area of waterlogging-treated Pakchio seedlings^31^. Also, exogenous GABA was reported to increase the dry weight of maize under hypoxia stress^48^. In our study, compared with WL treatment, GABA application improved the tolerance of maize seedling to waterlogging stress, showing that GABA application increased the plant height, leaf area, and biomass at two treated growth stages of maize cultivars XK-6 and ZD-958 (Table 1). Previously, studies found that GABA-induced improvement in many plant species could be due to improved photosynthetic activities, relative water content, osmolyte accumulation, leaf turgor, and other related physio-metabolical mechanisms^36,49,50^. GABA application might have improved the maize seedlings’ growth by inciting cell elongation and division and/or by maintaining metabolic balance within plant tissues. The results of the current study further confirm the beneficial effects of GABA application in maize under waterlogging conditions. Studies examining the physiological mechanisms underpinning the regulation of GABA on waterlogged maize seedlings are further needed.

Previous studies have found that the chloroplasts of plants subjected to abiotic stress are a major source of ROS, such as O_2_^−^ and H_2_O_2_, which are generated by the direct transfer of the excitation energy from chlorophyll or by oxygen reduction in the Mehler reaction^51,52^. In turn, the overproduction of ROS leads to lipid peroxidation and accumulation of MDA^53^. Our study indicated that waterlogging stress activated the production of H_2_O_2_ content, OH^−^ content, and O_2_^−^ content in maize seedlings, when compared with CK (Fig. 3A,C,D). The level of enhancements of these ROIs also coincided with a higher lipid peroxidation rate, which is regarded as a biochemical maker for free radicals mediated by the plant’s injury (Fig. 2A). These results are consistent with previous studies that examined the increments of ROIs production and MDA content under waterlogging stress^1,17,19,23,24,26,28–31,34–38,41–43,46,48–54^. The results of the present experiment showed that GABA treatment significantly decreased MDA content in both maize cultivars relative to the WL treatment (Fig. 3A,B), indicating that the application GABA effectively ameliorated the damages of waterlogging stress on the cell membrane system. It was also proposed that exogenous GABA was efficient at reducing the oxidative damage to cell membranes in melon seedlings under hypoxic conditions^55^, and tomato seedlings under chilling stress^42^. In the present study, we found that GABA might be involved in mitigating lipid peroxidation under waterlogging stress, resulting in a significant decrease in the accumulation of OH^−^, H_2_O_2_, and O_2_^−^. Our results showed that oxidative stress induced by waterlogging stress and damage to maize seedlings were effectively alleviated after exogenous GABA application. Previously, the beneficial effects of GABA have been observed under abiotic stress in maize seedlings^28,38,39,44–46,48,51,54–56^.

Commonly, photosynthetic apparatus, mitochondrial respiration, and photorespiration are considered as the main sources for generating ROIs under control conditions. Nevertheless, some other sources, such as XOD and MAO, also contribute to an active production of ROIs under abiotic stress^57^. The MAO, a flavoprotein localized on the outer membrane of mitochondria, catalyzes the oxidative deamination of aromatic amines and produces a large quantity of H_2_O_2_, which ultimately contributes to an increase in the steady state concentrations of ROIs within the plant cell^58^. Likewise, XOD can generate toxic ^⋅^O_2_^−^ as well as H_2_O_2_ in the plant cell and sometimes high production of ^⋅^O_2_^−^ from a XOD system can cause epidermal cell death that cannot be prevented by plant defense system^59^. In the current study, the activities of ROIs-producing enzymes of MAO and XOD were significantly higher under waterlogging stress treatments but remained lower under control conditions at different growth stages (Fig. 4). These results are in line with previous research, which found that the abiotic stress increased the activities of MAO and XOD in rice seedlings^60^. The activities of these two enzymes were concomitant with the level of ROIs in maize cultivars (Figs 3 and 4). For instance, MAO and XOD activities were significantly higher under waterlogging treatment, which led to higher levels of ROIs production. However, MAO and XOD activities were significantly lowered in plant leaves treated with the GABA application (Fig. 4), which might have caused the down-regulation of ROIs accumulation and oxidative stress in maize seedlings (Figs 3 and 4).

Previous studies reported that the SOD, APX, POD and GR enzymes of the plant antioxidative defense system were triggered under water stress^61,62^. The results of the present experiment showed that the activities of SOD, POD, CAT, APX, and GR activities significantly increased under waterlogging stress relative to that under CK in both stages (Fig. 5). It also has been reported that waterlogging significantly increased the activity of enzymatic antioxidants in maize leaves under waterlogging stress^62,63^. SOD is considered a main antioxidant enzyme that provides the first line of defense against ROIs because it catalyzes the dismutation process of ^⋅^O_2_^−^ to H_2_O_2_^64^, while CAT, POD, and APX help to remove H_2_O_2_ by converting it to oxygen and water^65,66^. Interestingly, exogenous GABA application significantly improved the activities of these five antioxidative enzymes at both stages of two varieties in two years compared with CK and WL treatments (Fig. 5). Increased activity of antioxidant enzymes in maize seedlings under GABA application is in agreement with previous studies on waterlogging stress in wheat^35^. These findings indicate that exogenous GABA application protected maize seedlings from oxidative stress caused by excessive water through the inhibition of lipid peroxidation, decreasing ROI levels, and activating antioxidant enzyme defense systems (Figs 3 and 5). Several studies have documented that exogenous GABA stimulates activities of antioxidant enzymes and reduces oxidative stress under different environmental stressors^30,35,36^. However, among these antioxidant enzymes, CAT, GR, and SOD were significantly activated by GABA application, with increases of 42.7%, 35.5%, and 32.2%, respectively, compared with those under WL treatment (Fig. 5C,D,A). Additionally, generation of ROIs often caused membrane damage and disintegration of various cellular structures and organelles, ultimately causing cell death^66–69^. However, SOD and POD acted against ROIs in GABA-treated maize seedlings to protect membrane damage, showing that GABA can potentially maintain cell integrity. GABA-induced maintenance of higher antioxidant activity is crucial to improve a plant’s ability to tolerate oxidative stress. Further research needs to be conducted to understand the molecular mechanism by which GABA regulates these antioxidant enzymes.

Similar to plant growth attributes, photosynthesis is one of the primary processes affected by waterlogging stress. Leaf gas exchange parameters of maize seedlings at V3 and V5 stages of two varieties in two years were significantly hindered by waterlogging stress (Fig. 2). These results are in agreement with findings from previous studies^1,46^. It was reported that the decline of Pn induced by stress was triggered by stomatal closure, which leads to inhibition of ambient CO_2_ diffusion to the mesophyll^69^. A similar phenomenon was observed in this study, where declines in Gs and Ci occurred and led to reduction in Pn. Meanwhile, our current results (Table 2) are in accordance with recent studies that show waterlogging stress causes reductions in the chlorophyll content in maize seedlings^1,46^. Nevertheless, application of GABA alleviated waterlogging stress on gas exchanges parameters in most experimental conditions in this study, when compared with WL treatment in both maize cultivars (Fig. 2). Our results showed that exogenous application of GABA on maize leaves after waterlogging could potentially retard declines in leaf chlorophyll content caused by waterlogging stress, resulting in the enhancement of photosynthetic characteristics (Table 2). Enhancements of photosynthetic characteristics in response to GABA application on leaves have also been observed in Pakchio seedlings under waterlogging stress^31^, and pepper seedlings under low light stress^29^. Exogenous GABA application was further reported to enhance the photochemical efficiency of PSII in muskmelon seedlings under hypoxia stress^37^. It was also reported that GABA can be transformed into succinic acid via the catalysis of γ-aminobutyrate transaminase and succinate semialdehyde dehydrogenase^45^. Succinic acid is then involved in the tricarboxylic acid cycle to maintain the carbon–nitrogen cycle in plants, which is known as the GABA metabolic bypass^70^. GABA application resulted in improvements in net photosynthesis in maize seedlings, possibly due to maintenance of cell turgor, which promoted chlorophyll biosynthesis (Table 2) and reduced oxidative damage by regulating various physio-biochemical processes^35–37^.

Decreases in chlorophyll content and photosynthetic efficiency were mostly caused by the disturbance of chloroplast morphology and ultrastructure of functional leaves^20,22^. Chloroplasts are major sites for generating reactive oxygen species (ROS) under environmental stress conditions^18^. However, present studies showed that waterlogging damaged leaf chloroplast ultrastructure, the number of chloroplast and grana number per chloroplast were reduced in response to the disintegration in a portion of the granum and reorganization of the chloroplast, the integrity of ultrastructure of chloroplast was demolished, their membranes and thylakoids were deliquescent in both maize seedling (Figs 7G–10G and 12G–14G and Table 2). Leading to reduced chlorophyll content and photosynthetic assimilation capacity^22,71^. At the same time, the chloroplast ultrastructure might be affected or damaged by waterlogging stress resulting from ROIs accumulation, because chloroplasts are a major source of active oxygen in plant tissue^18,72^. However, GABA application obviously increased the number of chloroplasts and grana compared with the waterlogging treatment (Table 2). In GABA treated plants, the chloroplast structure became a normal oval shape and grana were clear and visible (Figs 7A–9A,12A and 14A). However, the plant treated with exogenous GABA application increased the size of chloroplast and number of grana per chloroplast as compared with waterlogging treatment in both maize verities (Table 2). These results show that GABA application enhanced photosynthetic assimilation capacity, possibly because of an alleviation of chlorophyll degradation and preservation of ultrastructure. It was previously observed that exogenous 6-BA application enhanced chlorophyll content and alleviated the degeneration of photosynthetic performance caused by waterlogging stress in maize seedlings^42^. However, GABA application has not been previously shown to have an effect on the ultrastructure of chloroplasts, and further studies are needed to clearly understand the mechanisms at the transcriptional molecular and gene level.

In summary, results of our study suggest that exogenous GABA application could enhance the growth of maize seedlings under waterlogging stress through the activation of antioxidative enzymes, alleviation of ROIs impacts, mitigation in damage on leaf chloroplast ultrastructure, and enhanced photosynthesis. Findings of this study provide insights into the physiological mechanisms underlying GABA-induced acclimation of maize to waterlogging stress and provides a possible solution to manage waterlogged maize at an early stage in the field.

## Materials and Methods

### Plant culture and experimental design

The experiment was conducted in 2016 and 2017 in a greenhouse to avoid the influence of rainfall on soil water treatment at the Huazhong Agricultural University in Wuhan, China (30°47′N, 114°35′E). Two maize hybrids commonly found in China were used in this study: Zhengdan-958 (ZD-958) and Xing Ken-6 (XK-6). The experiments were laid out in a randomized design with three replications. Before sowing, the healthy seeds of both maize varieties were sterilized by soaking in 1% (v/v) sodium hypochlorite for 30 min and then kept in the incubator for germination at 28 °C in darkness for about 3 d. Uniformly germinated seeds were selected and sown in soil in pots that had been prepared 10 d prior to the experiment and had already reached the appropriate soil water content for maize emergence. Six germinated seeds were sown in plastic pots (32 cm lower inside diameter, 34 cm upper inner diameter, 30 cm height); these pots were filled with 20 kg of sieved dry soil amended with 0.14 g urea, 0.14 g diammonium phosphate, and 0.18 g potassium chloride per kg soil and seedlings were thinned to three plants per pot at the one-leaf (V1) stage. Each treatment included 12 pots for each growth stage and each variety with three replicates. All measures against diseases and insect infestation of the maize seedlings were deployed at the appropriate time during the experimental period.

To examine the effects of GABA application on the growth of maize seedlings, waterlogging was initiated at third leaf stage (V3) and fifth leaf (V5) stages in both years. The pots intended for the waterlogging treatments were filled with water to 1–2 cm above the soil surface for 14 d. Different concentrations of GABA (0.25 mmol L^−1^, 1.00 mmol L^−1^, and 1.75 mmol L^−1^) were sprayed on plant leaves during waterlogging in 2016. Based on plant growth, chlorophyll content, and photosynthesis data in 2016 (Table S1), one optimal concentration of GABA (1 mmol L^−1^) was chosen and used in the validating experiments in 2016 and 2017, with same regime as described above. The waterlogged plants were sprayed three times with GABA (GABA treatment) 1 d, 3 d, and 6 d after waterlogging implementation. To prevent contamination to other treated plants, non-targeted pots were fully isolated with plastic film every time the plants were sprayed. A randomized complete block design was applied with three replications. Meanwhile, two control treatments were carried out each year, including normal soil moisture control treatment (CK) and waterlogging control treatment without spraying GABA (WL). Representative maize seedlings under different treatments at the V5 stage are shown in Figure S1.

### Plant sampling and measurements

Twelve representative plants of each treatment were carefully removed from 4 selective pots for each variety at V3 and V5 stages at the end of the waterlogging period (14 d) and then separated into root parts and shoot parts. An upmost fully expanded young leaf was selected from three plants per replicate and leaves were quickly stored at −80 °C for physiological indicator analysis. Six plants from each treatment were rapidly transferred to ovens, dried at 105 °C for 30 min, and then dried at 80 °C to a constant mass and weight for dry matter determination. Growth was determined in terms of plant height, stem diameter, green leaf area, and dry weight. Green leaf area (GLA) was calculated according a method published previously^73^.

### Measurement of Endogenous GABA Content

The endogenous GABA content was determined by Berthelot reaction with some modifications^74^. Leaves (0.1 g) were ground with methanol at room temperature. The homogenate was centrifuged at 5,000 g for 15 min and discarded the supernatant (2–3 times). The sediment was re-dissolved in 1.5 mL distilled water. Subsequently, the samples were heated in water bath at 50 °C for 2 h, and then centrifuged at 7,000 g for 15 min. One milliliter supernatant was added 0.1 mL 2 mol/L AlCl_3_ and oscillated. The mixture was cooled to room temperature and then centrifuged at 12,000 g for 10 min. The supernatant (0.5 mL) was shaken for 5 min with 0.3 mL KOH and centrifuged at 12,000 g for 5 min. The resulting supernatant was used to measure the content of GABA based on the following procedure: 0.3 mL supernatant was added to the reaction solutions [including 0.5 mL 0.1 mol/L sodium tetraborate (pH = 10.0), 0.4 mL 6% phenol and 0.6 mL 5% sodium hypochlorite]. The mixture was put into a boiling water for 10 min and rapidly placed in ice bath for 5 min. Finally, the solution was shaken with 2 mL 60% ethyl alcohol and measured the absorbance in 645 nm. The endogenous GABA content was calculated using a standard curve.

### Gas exchange parameters and chlorophyll contents

Gas exchange parameters such as the net photosynthetic rate (Pn), stomatal conductance (Gs), intercellular CO_2_ (Ci), and transpiration rate (Tr) were measured in both years between 10:00 and 12:00 h, by using a LI-6400 portable photosynthesis system (LI-COR Inc., Lincoln, NE, USA) during three (V3) and fifth leaf (V5) stage of both maize varieties. Chlorophyll (Chl) contents were quantified by using the method^75^. Chlorophyll contents were extracted from 0.1 g leaf discs with 8 mL acetone (80%) and kept in dark conditions for 24 h. The absorbance of the supernatant was measured at 646 and 663 nm using spectrophotometer (UV2102; Unico, Shanghai, China).

### Sample preparation and observation by Transmission Electron Microscope (TEM)

For leaf tissue preparation and electron microscopy, small leaf discs (4.0 mm × 1.2 mm) from within the gas exchange chamber were removed and infiltrated in a syringe with the fixative 2.5% glutaric aldehyde in 0.1 m phosphate buffer (pH = 7.6) at 4 °C, and post-fixed in 2% buffered osmium tetroxide at 20 °C for 2 h. The samples were embedded in Spurr’s epoxy resin (Sigma-Aldrich, St. Louis, USA). For light microscopy, semi thin leaf cross sections were stained with toluidine blue, and observed at 200× magnification with an Olympus IX71 light microscope (Olympus Optical, Tokyo, Japan). Ultrathin leaf cross sections were stained with 4% (w/v) uranyl acetate followed by 2% (w/v) lead citrate. Transmission electron microscope (H-7650; Hitachi – Science &Technology, Tokyo, Japan) and Soft Imaging System software (H-7650; Hitachi –Science & Technology, Tokyo, Japan) were used for observation and photography. The width and length of chloroplast was measured by software Image J, a free, Java-based image-processing package.

### Determination of membrane lipid peroxidation, contents of H_2_O_2_, superoxide (O_2_) and contents of hydroxyl ion (OH^−^)

The biochemical analyses were carried out in the fresh leaves samples. MDA determination kit (A003-1), (Nanjing Jiancheng Bioengineering Institute, NJBI) according to the manufacturer’s instructions. Briefly, the leaf tissue homogenate was centrifuged at 12000 g and 4 °C for 15 min, and the supernatants were collected. MDA and thiobarbituric acid (TBA) mixture was produced during the reaction of MDA in samples with TBA, and then this mixture was measured at 535 nm and final result of MDA was expressed as mg^−1^ protein. H_2_O_2_ was extracted and its content was measured by monitoring the absorbance of the titanium-peroxide complex at 405 nm according to the method of Nanjing Jiancheng Bioengineering Institute (NJBI) determination kit. The contents of H_2_O_2_ were demonstrated as unit mg^−1^ protein. The contents of hydroxyl ion (^⋅^OH^−^) and superoxide anion radical (^⋅^O_2_^−^) in the leaves of maize seedlings were determined using the commercial OH^−^ assay kit (A018) and O^⋅−^_2_ assay kit (A052), respectively, obtained from Nanjing Jiancheng Bioengineering Institute, China. The ^⋅^OH^−^ was expressed as units mg^−1^ protein, and one unit was the amount required to reduce 1 M of H_2_O_2_ in the reaction mixture per minute at 37 °C. The ^⋅^O_2_^−^ were demonstrated as units g^−1^ protein, and one unit was equivalent of the value required to inhibit superoxide anion by 1 mg of VC for 40 min at 37 °C.

### Assay of Monoamine oxidase and Xanthine oxidase contents

The activities of reactive oxygen species -producing enzymes viz., XOD and MAO were measured by using the commercial kits in accordance with manufacturer’s instruction (Nanjing Jiancheng Bioengineering Institute, NJBI). The XOD contents was assayed by determination kit A034 (NJBI). The XOD was defined as 1 g of protein required transform as 1 µM of hypoxanthine to xanthine formed per minute at 37 °C. The activities of XOD enzymes were expressed as 1 unit mg^−1^ protein. The MAO contents was assayed by determination kit A002 (NJBI). The MAO was defined as the amount of enzyme that increased the absorbance by 0.01 at 37 °C in 1 hour; 1 unit g^−1^ protein. The activities of MAO enzymes were demonstrated as 1 unit mg^−1^ protein.

### Assay of antioxidant enzymes

The antioxidants enzymatic were analyses by using the commercial kits in accordance with the manufacturer’s guidelines. The kits for antioxidants enzymatic were purchased from Nanjing Jiancheng Bioengineering Institute (NJBI). The SOD activity was assayed by determination kit A001 (NJBI). One unit of SOD activity was defined as the amount of enzyme required for 1 mg tissue protein in 1 ml of reaction mixture SOD inhibition rate to 50% as monitored at 550 nm. The activities of SOD were demonstrated with unit mg-1 protein. The determination of POD activity was determined with the assays kit A084-3 (NJBI). One unit of POD activity was defined as the amount of enzyme necessary for the decomposition of 1 μg substrate in 1 minute at 37 °C. The POD activities were expressed with unit mg^−1^ proteins. CAT activity was assayed by determination kit A007-2 (NJBI). One unit of CAT activity was defined as 1 mg tissue protein consumed 1 µmol H_2_O_2_ at 405 nm for 1 sec. The activities of CAT were demonstrated with units mg^−1^ protein. The determination of GR activity was determined with the assays kit A062 (Nanjing Jiancheng Bioengineering Institute). One unit of GR activity was defined as 1 g tissue protein consumed 1 mmol NADPH at 340 nm for 1 min. The activities of GR were demonstrated with units mg^−1^ protein. The determination of APX activity was determined with the assays kit A123 (NJBI). One unit of APX activity was defined as 1 mg tissue proteins catalysised 1μmol ascorbate at 290 nm for 1 min. The APX activity was demonstrated as units g^−1^ protein.

### Statistical Analysis

Analysis of variance (ANOVA) were performed using the software Statistix 10.0 (Analytical Software, Tallahassee, FL, USA) Statistically significant differences between control and GABA treatment samples were tested by Fisher’s least significant difference (LSD) test at a probability of P < 0.05.

## Electronic supplementary material

Supplementary FILE
