# Supplementary material for: γ-Aminobutyric Acid Promotes Chloroplast Ultrastructure, Antioxidant Capacity, and Growth of Waterlogged Maize Seedlings
Source: Sci Rep. 2019 Jan 24;9:484. doi: 10.1038/s41598-018-36334-y (PMC6345989; doi:10.1038/s41598-018-36334-y)
Supplement: Supplementary file 1 — Supplementary FILE [file 41598_2018_36334_MOESM1_ESM.docx]

**γ-Aminobutyric Acid Promotes Chloroplast Ultrastructure, Antioxidant Capacity, and Growth of Waterlogged Maize Seedlings**

**Akram Salah^1^, Ming Zhan^1*^, Cougui Cao^1^, Han Yuling^1^, Lin Ling^1^, Zhihui Liu^1^, Ping Li^1^, Miao Ye^1^ and Yang Jiang^1^**

1 MOA Key Laboratory of Crop Physiology, Ecology and Cultivation in the Middle Reaches of Yangtze River, College of Plant Science and Technology, Huazhong Agricultural University, Wuhan, China, 430070


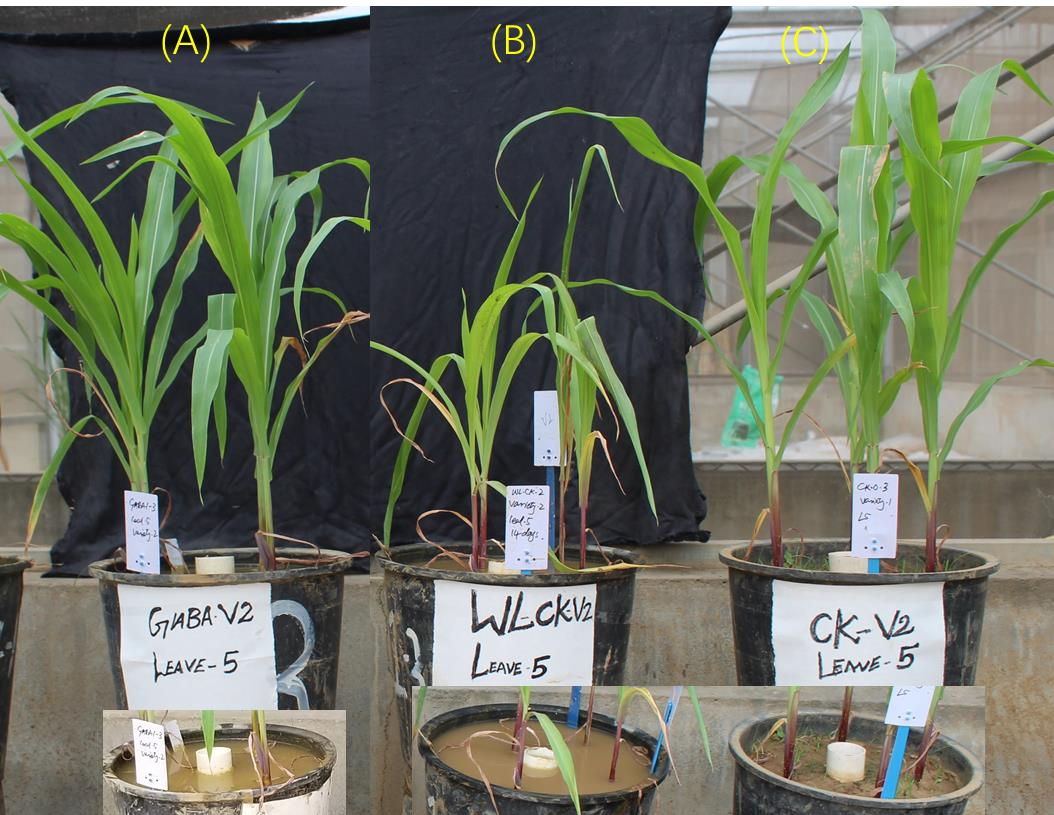


**Figure S1** Photos of representative maize seedlings under GABA treatment with waterlogging condition (A), the waterlogging control treatment (B) and the normal soil water control treatment (C) which were initiated at the fifth leaf stage.

**Table S1** Effects of exogenous GABA application with different concentration on Chlorophyll content, growth attributes of XK-6 and ZD-958 maize seedlings after 7 d waterlogging stress initiated at 3-leaf (V3) and 5-leaf (V5) stage in 2016

|  |  | **Chl content** | |  | **Plant Height** | |  | **Aboveground DM** | |  | **Root DM**  **(g/ plant^-1^)** | |
| --- | --- | --- | --- | --- | --- | --- | --- | --- | --- | --- | --- | --- |
|  |  | **(mg g-^1^ FW)** | |  | **(cm)** | |  | **(g/ plant-^1^)** | |  |  |  |
| **Stages** | **Treatment** | XD-6 | ZD-958 |  | Ken-6 | ZD-958 |  | Ken-6 | ZD-958 |  | Ken-6 | ZD-958 |
| **V3** |  |  |  |  |  |  |  | |  |  |  |  |
| GABA ( 0.25 mmol/ L^-1^) | | 1.18 c | 0.98 cd |  | 32.7b | 29.5 cd |  | 0.74 b | 0.56 bc |  | 0.30 bc | 0.31 c |
| GABA ( 1mmol/ L^-1^ ) | | 1.38 b | 1.46 b |  | 33.5 b | 32.9 b |  | 0.82 b | 0.84 b |  | 0.35 b | 0.36 b |
| GABA (1.75 mmol/ L^-1^) | | 1.17 bc | 1.09 c |  | 31.7 b | 29.8 c |  | 0.74b | 0.72 bc |  | 0.32 bc | 0.34 b |
| CK | | 1.81 a | 1.93 a |  | 40.2 a | 38.7 a |  | 1.46 a | 1.60 a |  | 0.84 a | 0.74 a |
| WL | | 0.88 d | 0.84 d |  | 28.8 c | 26.7 d |  | 0.65 bc | 0.62 bc |  | 0.25 c | 0.26 c |
| **V5** |  |  |  |  |  |  |  |  |  |  |  |  |
| GABA ( 0.25 mmol/ L^-1^) | | 1.11 d | 1.22 d |  | 54.3 c | 49.9 bc |  | 2.28 b | 2.17 c |  | 0.65 c | 0.60 c |
| GABA ( 1mmol/ L^-1^ ) | | 1.57 b | 1.69 b |  | 58.5 b | 54.6 b |  | 2.44 b | 2.42 b |  | 0.74 b | 0.76 b |
| GABA (1.75 mmol/ L^-1^) | | 1.63 b | 1.47 c |  | 55.8 c | 56.5 b |  | 2.30 b | 2.29 bc |  | 0.72 bc | 0.72 b |
| CK | | 2.09 a | 2.01 a |  | 67.7 a | 64.3 a |  | 3.90 a | 3.70 a |  | 1.34 a | 1.22 a |
| WL | | 1.25 c | 1.17 d |  | 52.1 c | 46.1 c |  | 1.95 c | 1.89 d |  | 0.53 d | 0.56 c |

Values followed by a different small letter within a column are significantly different at 5% probability level for comparison among different treatments at same growth stage.
